# In or Out-of-Madagascar?—Colonization Patterns for Large-Bodied Diving Beetles (Coleoptera: Dytiscidae)

**DOI:** 10.1371/journal.pone.0120777

**Published:** 2015-03-20

**Authors:** Rasa Bukontaite, Tolotra Ranarilalatiana, Jacquelin Herisahala Randriamihaja, Johannes Bergsten

**Affiliations:** 1 Department of Zoology, Swedish Museum of Natural History, Box 50007, Stockholm, Sweden; 2 Department of Zoology, Stockholm University, Stockholm, Sweden; 3 Departement d’Entomologie, Faculté des Sciences, B.P. 906, Université d’Antananarivo, Antananarivo, Madagascar; 4 Programme National de Lutte contre le Paludisme de Madagascar, Androhibe, Antananarivo (101), Madagascar; University of Stellenbosch, SOUTH AFRICA

## Abstract

High species diversity and endemism within Madagascar is mainly the result of species radiations following colonization from nearby continents or islands. Most of the endemic taxa are thought to be descendants of a single or small number of colonizers that arrived from Africa sometime during the Cenozoic and gave rise to highly diverse groups. This pattern is largely based on vertebrates and a small number of invertebrate groups. Knowledge of the evolutionary history of aquatic beetles on Madagascar is lacking, even though this species-rich group is often a dominant part of invertebrate freshwater communities in both standing and running water. Here we focus on large bodied diving beetles of the tribes Hydaticini and Cybistrini. Our aims with this study were to answer the following questions 1) How many colonization events does the present Malagasy fauna originate from? 2) Did any colonization event lead to a species radiation? 3) Where did the colonizers come from—Africa or Asia—and has there been any out-of-Madagascar event? 4) When did these events occur and were they concentrated to any particular time interval? Our results suggest that neither in Hydaticini nor in Cybistrini was there a single case of two or more endemic species forming a monophyletic group. The biogeographical analysis indicated different colonization histories for the two tribes. Cybistrini required at least eight separate colonization events, including the non-endemic species, all comparatively recent except the only lotic (running water) living *Cybister operosus* with an inferred colonization at 29 Ma. In Hydaticini the Madagascan endemics were spread out across the tree, often occupying basal positions in different species groups. The biogeographical analyses therefore postulated the very bold hypothesis of a Madagascan origin at a very deep basal node within *Hydaticus* and multiple out-of-Madagascar dispersal events. This hypothesis needs to be tested with equally intense taxon sampling of mainland Africa as for Madagascar.

## Introduction

Madagascar has been isolated for 130–160 Ma from mainland Africa and 80–90 Ma from India [1]. Being the fourth largest island on Earth, it presents five biomes with unique vegetation compositions [2], providing ample opportunities for endemic radiations [3]. The level of endemism on Madagascar is unmatched by any other similar-sized area: 83% of plants, 99% of amphibians, 92% of reptiles, 52% of birds, 93% of freshwater fishes, 100% of terrestrial mammals and 86% of invertebrates [4]. This high rate of endemism typically occurs not only at species level, but also at higher taxonomic levels, i.e. 23 out of 24 genera and 1 out of 4 families of amphibians are endemic to Madagascar [3].

High species diversity and endemism on large oceanic islands is often the result of radiations following vicariance events or colonizations from nearest continents or other islands [5]. Numerous endemic clades of Malagasy taxa arrived from mainland Africa, but some seem to have their origins in Asia or even South America [1, 6, 7]. Speciation due to continental vicariance events related to the break-up of Gondwana are presently limited to few potential examples of sufficient age such as freshwater crayfish [8, 9]. The vast majority of studies report that endemic species have their origins in later colonizations of the island, sometime during the Cenozoic, when Madagascar was already surrounded by sea [1, 10, 11]. Moreover, it is commonly found through phylogenetic reconstruction, that endemic diversity on Madagascar can be traced back to single or a small number of colonization events. The classical example of this for terrestrial mammals includes the flagship animals, and largest tourist attraction on Madagascar, the lemurs. Lemurs, tenrecs, rodents and even carnivorans, previously classified as felids and mongoose, can each have their origin traced back to divergence on the island after single asynchronous colonization events [1, 12–14].

While mammals, birds, amphibians and reptiles have been the main focus of much of the research into colonization and speciation on Madagascar, studies on the hugely diverse insects are starting to appear. It has been reported that 90–100% of Malagasy insect species are endemic to the island, but share many family and genus level affinities with mainland Africa [4]. Similar to vertebrates, most analyses suggest Cenozoic transoceanic dispersal events in various insect groups. For example, studies on dung beetles [15] showed Late Cenozoic origins followed by several *in situ* radiations [16–18]. Malagasy carpenter bees exhibit several dispersal events from Africa to Madagascar ranging from 25 to 9 Ma [19], and for the Malagasy Allodapine bee clade colonizations were inferred to be around 40–43 Ma [20, 21]. Pierid butterflies suggest that major diversification in *Colotis* occurred in Africa with subsequent dispersal to India and Madagascar [22]. Also mayflies were reported to have colonized Madagascar multiple times [23]. However fungus-growing termites of Madagascar all originated from a single colonization event at 13 Ma [24].

Although the majority insects groups have wings and are capable of flight, their ability for long distance flight varies immensely. Some dragonflies, butterflies and Dipterans are true migrants and can traverse thousands of kilometers following trade winds and favorable air currents [25]. Other winged insects such as species of mayflies, caddisflies, and stoneflies are capable of flight but rarely fly more than 100 meters lateral to their place of birth [26]. This difference in behavior and flight capacity naturally has an effect on the colonization potential of oceanic islands. Similar to terrestrial mammals, insects also could have rafted on blocks of vegetation washed out from river deltas, utilizing favorable sea currents during parts of the Cenozoic, to cross the Mozambique channel and arrive on the shores of Madagascar in some 25 days [27]. More importantly, flight capacity may have a crucial influence on what happens after a successful colonization. What level of flight capacity is sufficient for upholding geneflow across an island the size of Madagascar, preventing further speciation? At what level of flight capacity do the larger rivers constitute dispersal barriers capable of giving rise to speciation and the type of microendemic distribution pattern seen in many groups [28]? These questions have so far been poorly explored when it comes to the origin and diversification of endemic insects on Madagascar. Time-calibrated phylogenetic species-level analyses may not only answer the question of when and how many times Madagascar was colonized, but also the fate of the arrivals, their dispersal capacity relative to island size and its influence on geneflow, panmixis or *in situ* diversification.

**Fig 1:**
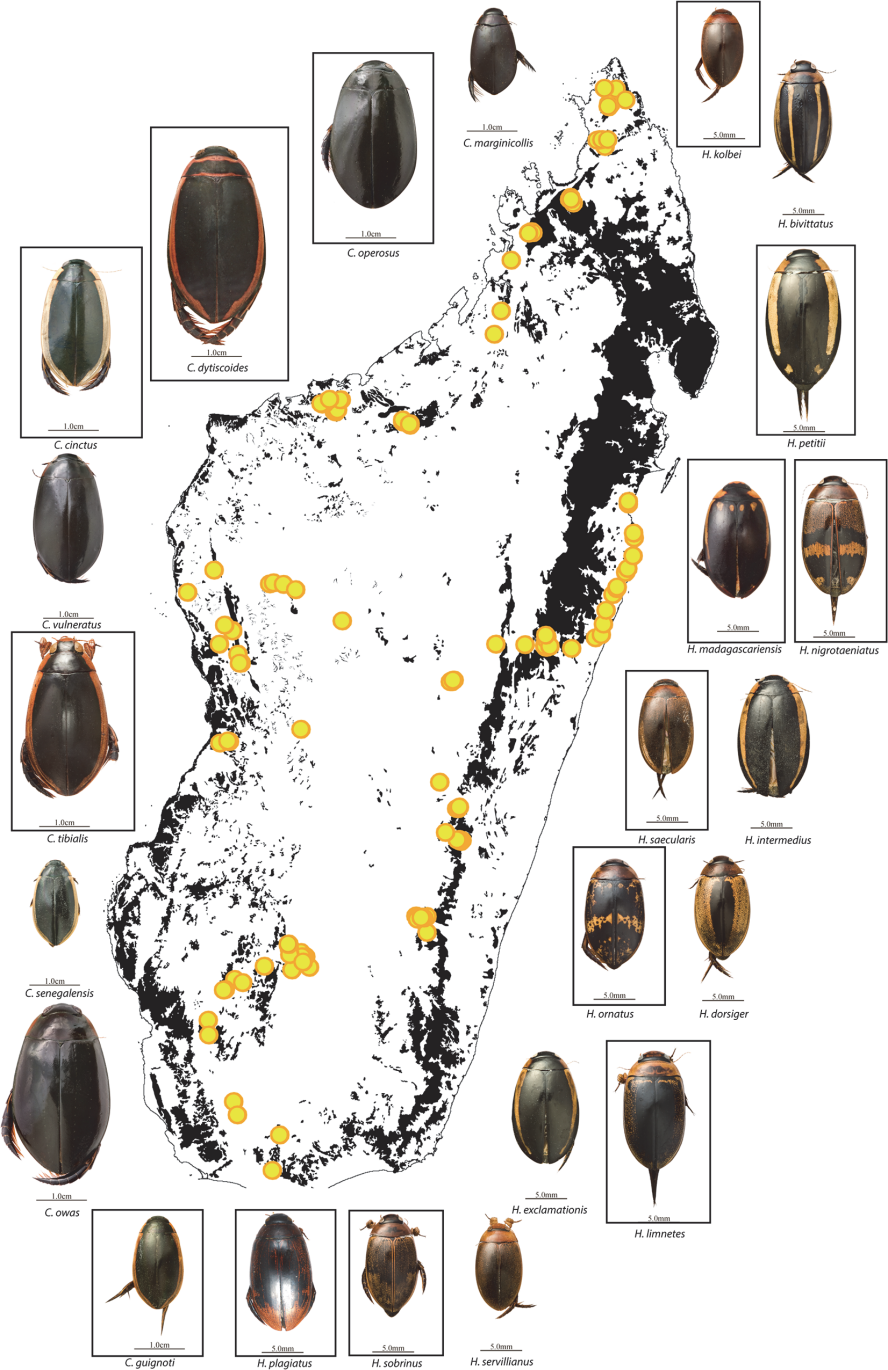
Collecting localities from four expeditions 2006–2012 across the island and known species of *Cybister* and *Hydaticus* on Madagascar. Species in frames represent species that are endemic to Madagascar. Photos by Harry Taylor and Johannes Bergsten.

The colonization and speciation history of freshwater beetles on Madagascar is largely unknown. This species-rich group is often a dominant part of smaller freshwater communities and is important as indicator for freshwater biodiversity assessment [29]. Freshwater habitats are the most threatened ecosystems on earth and conservation efforts for the remaining natural aquatic habitats of Madagascar are badly needed [30–32]. Water beetles in Madagascar range from species less than a millimeter in length to almost four centimeters, absolute flight capacity often being correlated with body size. The group contains numerous species, both endemic and non-endemic, as well as some endemic genera with species radiations. Here we focus on the large bodied diving beetles (Dytiscidae) of the tribes Hydaticini and Cybistrini. Both tribes have a worldwide distribution [33] and inhabit mostly standing waters but also slower or protected parts of running waters [34]. Adults generally have good flight capability and can be found in both temporary and permanent water bodies [35]. However, for most species, documentation of actual observed flight is lacking. Both tribes have recently been the focus of phylogenetic analysis on a global scale [34, 36]. These phylogenies provide the global framework necessary to test the origin, timing and number of colonization events to Madagascar. By sampling all Malagasy species in these tribes and include them in these frameworks we can answer the questions of when, from where, and how many times has Madagascar been colonized. Fourteen species of Hydaticini and nine species of Cybistrini are known from Madagascar [37], but many are very rare and only known from the type localities, or old historical records. Here, after four expeditions 2006–2012 across the island, we have sampled fresh material for 21 of the 23 known species (Fig. 1), and attempted to extract DNA from the remaining two species from historical museum material. Our aim is to answer the following questions 1) How many colonization events do the present Malagasy species originate from? 2) Did any colonization event lead to a species radiation? 3) Where did the colonizers come from and have there been any out-of-Madagascar events? 4) When did these events occur and were they concentrated to any particular time interval?

## Materials and Methods

### Taxon sampling

Collecting was done under permits provided by Ministère de l’Environment et des Forets: No.82/06/MINENV.EF/SG/DGEF/DPB/SCBLF/RECH (2006), No.250/06/MINENV.EF/SG/DGEF/DPB/SCBLF/RECH (2006–2007), No266/09/MEF/SG/DGF/DCB.SAP/SLRSE (2009), No250/11/MEF/SG/DGF/DCB.SAP/SCB (2011–2012). We used GB water nets, bottle traps and crayfishtraps, the latter two baited with fish, liver, catfood or lightsticks. Specimens were preserved in the field in 95% ethanol and later transferred to absolute ethanol and stored in -20°C freezers. Tissue from large specimens of Cybistrini were either extracted from the thorax region already in the field, or ethanol was injected to the hameolympha post-mortem with a syringe, as DNA degradation otherwise can occur due to the slow permeability of their exoskeleton and replacement of bodyfluids with ethanol. Several of the non-endemic species, like *Hydaticus dorsiger*, *H*. *exclamationis*, *H*. *bivttatus*, *H*. *servillianus*, *Cybister senegalensis*, and *Cybister vulneratus* were found in degraded antropogenically disturbed habitats. The endemic *Hydaticus nigrotaeniatus*, *H*. *sobrinus* and *H*. *petitii* were found in several natural habitats of rainforest at midaltitude. The endemic species *Cybister tibialis* was only found in 2009, in an area of the drier western deciduous region. Endemic *Hydaticus ornatus* was found in some numbers in deep canyons of the Isalo National Park in the southwest. Endemic *Hydaticus madagascariensis* was only known from historical records in the Paris museum without detailed locality information, but a few specimens were found in 2012 on the lowland eastcoast. Endemic *Hydaticus limnetes* and *H*. *saecularis*, only known from the type material was found in numbers by tramping the vegetation at a forest swamp without visible surface water in Ranomafana National Park (midaltitude rainforest).

The only two known species we have not managed to find are *Hydaticus plagiatus* and *Cybister dytiscoides*. *Hydaticus plagiatus* is only known from the unique type specimen in the Paris museum, without any locality detail except Madagascar. *Cybister dytiscoides* is the largest diving beetle species on Madagascar and is known from a few specimens only. These large diving beetles are fast swimmers and difficult to catch with a hand net why we have used baited bottle traps or crayfish traps overnight at numerous localities. With these we have managed to collect the other large *Cybister* species but unfortunately not *C*. *dytiscoides*. Like with other non-recovered known species after extensive inventory, it is not unlikely that they are extinct, especially when the only known localities are correlated with forest loss since they were discovered [38]. A recent collecting expedition in late 2014 to the northeast area (Marojejy NP and Anjanaharibe-Sud NP, not marked in Fig. 1) also failed to find these species and neither recovered any new species from the target groups. Screening of potential cryptic species using large throughput sequencing of CO1 for multiple individuals per species has not found any new potential species of Hydaticini or Cybistrini apart from in one case [30, 39]. The endemic *Hydaticus nigrotaeniatus* which occurs throughout the eastern rainforest belt from north to south vary in colour pattern and showed considerable genetic variation related to geography. But it has not yet been evaluated if the variation is a gradual distance-decay relationship or a discontinuous variation in this species why we here treat it as a single species.

We also added the afrotropical *Hydaticus dregei* as a representative of the *speciosus* species-group of *Hydaticus* as this was not represented in Miller et al. [34], and the Mauritius endemic *Cybister desjardinsii* for dating purposes (see below).

### Morphology

The previously published datasets for Hydaticini and Cybistrini included morphological character matrices of 36 and 47 characters respectively, mostly informative between genera and subgenera [34, 36]. Character states were scored for the added species and coded according to previous descriptions [34, 36, 40]. The morphological character matrices were analyzed with a markov k model [41] together with the molecular data.

### DNA sequence

DNA extraction, PCR and sequencing were done for the 21 species with freshly collected material following the protocol described in Bukontaite et al [42]. To include the species into the datasets compiled by Miller et al. [34, 36] we amplified and sequenced the same two mitochondrial and two nuclear genes as in these studies: cytochrome c oxidase subunit I and II (COI and COII), Histone (H3) and wingless (Wnt). For the last two known Madagascan species *C*. *dytiscoides* and *H*. *plagiatus* we attempted to extract degraded DNA from specimens borrowed from the Paris museum. After permission was given we carefully removed one hind leg and placed it in extraction buffer provided with DNEasy DNA extraction kit (Qiagen, Valencia, CA) following the manufacturer’s recommendation, except for that 20 μl of 1 molar DTT (dithiothreitol) was added during the lysis stage. After overnight incubation the hind leg was recovered intact and reglued to the specimen providing a near non-destructive DNA extraction of these valuable materials. We attempted to sequence short 100–200bp fragments of COI using newly designed primers: 5’ACT AAT GGA AAT GAG CAA CAA CA and 3’GAG CTT ATT TTA CTT CAG CAA CT. We were unable to amplify any DNA from the holotype of *H*. *plagiatus*, but for *C*. *dytiscoides* we managed to sequence a short authentic gene fragment. Sequences are submitted to NCBI GenBank under accession numbers KP280087-KP280171 (S1 Table).

Chromatograms were edited in Sequencher 4.10 (Gene Codes Corporation), contigs were created from forward and reverse reads and primer regions were removed. DNA sequences were aligned together with the sequences downloaded from Genbank from Miller et al. [34, 36] with MUSCLE [43] in MEGA 5 [44]. Data tables and matrices were created with Voseq 1.7.3 [45] (S1 Table).

### Phylogenetic analyses

We used Bayesian inference to estimate the phylogenetic placement of the Malagasy species to each other and to the species sampled worldwide in the two published studies by Miller et al. [34, 36]. In addition, we used Maximum Likelihood (ML) with bootstrap analysis to assess clade support values. As outgroups to root the trees we used the same representatives from related tribes in the subfamily Dytiscinae as used in previous datasets [34, 36]: *Thermonectus variegatus*, *Notaticus fasciatus*, *Dytiscus marginalis*, *Dytiscus verticalis* and *Hyderodes shuckardi*.

Miller et al. [34] tested the best partitioning scheme for datasets with multiple nuclear and mitochondrial genes using Bayes factors. They concluded that separating genome source (mitochondrial or nuclear) and codon position was significantly better than gene specific partitions. Therefore we analyzed both combined datasets with 7 partitions; 1st, 2nd and 3rd codon positions for nuclear and mitochondrial genes as separate partitions, and the morphological characters as the 7th partition. Analyses were conducted using RAxML-HPC BlackBox 8.1.11 [46] and MrBayes 3.2.2 [47] on Cyberinfrastructure for Phylogenetic Research (CIPRES) Science Gateway portal V 3.3. (without the BEAGLE option) (www.phylo.org/index.php/portal/).

The Bayesian analyses used the same model settings for the seven partitions as published in the original papers by Miller et al. [34, 36]. For Cybistrini this meant that each partition had a GTR+I+G model but that the substitution rate and state frequency parameters were linked across the nuclear and mitochondrial partitions, respectively, and that the gamma shape parameter was linked across the 1st and 2nd nuclear and mitochondrial codon partitions, respectively. For Hydaticini the partitions were given the models suggested by the Bayesian Information criterion as calculated by MrModeltest [48]: COI-COII_pos1: GTR+I+G, COI-COII_pos2: HKY+I+G, COI-COII_pos3: HKY+G, H3-Wnt_pos1: GTR+G, H3-Wnt_pos2: JC+G, H3-Wnt_pos3: HKY+G.

However the authors reported the possibility of an effect from long branch attraction between the species *H*. *nigrotaeniatus* and *H*. *parallelus* in the Hydaticini dataset [34]. For this reason, we changed the default branchlength prior to an exponential distribution with a mean of 0.01 (rate parameter = 100) in an attempt to eliminate biased branch lengths [49, 50].

In the Hydaticini analysis, for dating purposes (see below) we also needed to include the taxa from the sistergroup of Hydaticini, Aciliini+Eretini [42]. As Aciliini, Eretini and their sistergroup relationship was overwhelmingly supported by the eight gene dataset in Bukontaite et al. [42] we here included this as a prior and constrained them as monophyletic. Two parallel analyses for each datasets were run each with one cold and three heated chains for 10 000 000 generations with a sample frequency of 1000. A burn-in fraction of 25% was discarded and the remaining 7500 trees from both runs pooled before a majority-rule consensus tree was calculated.

Convergence and mixing was assessed using the statistics provided by the MrBayes program and by using Tracer v 1.5. [51]. The convergence of the topologies were tested by using the cumulative command in AWTY, which displays the posterior probabilities of splits over a MCMC run [52]. ML analyses were run with the GTRGAMMA model (for the best tree search) and GTRCAT model (for boostrap support) for each of the same partitions as above in RAxML BlackBox [53].

The final trees were visualized in FigTree 1.3.

## Dating

Calibrated trees were obtained by using the topology retrieved from MrBayes in BEAST 1.7.4. [51]. We used the same partitions as in the phylogenetic analyses and allowed each a separate substitution rate. The uncorrelated lognormal clock was used to relax the equal rates constraint across the tree [54]. For calibrating the trees we used different calibration points for Hydaticini and Cybistrini.

### Hydaticini

There are few fossil records of the Hydaticini tribe, all very recent and too young to be useful as calibration points [33]. For this reason we used the fossil record *of Acilius florissantensis* (age 34 Ma) as a crown-node constraint of *Acilius* following Bukontaite et al. [42]. The interpretation of the fossil as belonging to the crown node of *Acilius* was supported by vicariance-favoured biogeographical analyses, but see Bukontaite et al [42] for a discussion. The prior on the age of the node was set to a lognormal distribution with an offset at 34,0 Ma (mean = 7, logStd = 0,5), which gives 95% of the prior distribution between 36 and 50 Ma.

### Cybistrini

Again, few useful fossils are available for Cybistrini [33] and in addition Miller and Bergsten [36] showed that Cybistrini is not even closely related to Dytiscinae and were by the authors elevated to separate subfamily status. Instead, we tentatively used a geological event as calibration by including the Mauritius endemic species *Cybister desjardinsii*. Mauritius is a volcanic island with an estimated geological age of 7–8 Ma [55], which provides a potential maximum age for the endemic species *C*. *desjardinsii* (but see Heads [56] for a general critique of using island ages to date island endemics). We set up the prior for the age of the MRCA of *C*. *desjardinsii* and its sister with a lognormal distribution with stdev = 0,2 and mean = 1,8. This prior quantifies our prior beliefs that speciation occurred maximum 8 Ma but with maximum probability somewhat later around 5–7 Ma and with a decreasing probability towards present.

As this calibration point is very recent, we also used a deeper secondary calibration point between *Thermonectus* and *Dytiscus*, which was obtained from the analysis of Bukontaite et al. [42]. We used a normal distribution prior with mean = 112 and Stdev = 12, which covers the 95% age interval as estimated by the authors.

## Biogeography

The current distribution areas of species were obtained from Nilsson [33] but in some cases modified following Löbl and Smetana [57]; if e.g. Palearctic occurrence in Nilsson [33] is based on a marginal contact with the Palearctic zooregion in an otherwise entirely Afrotropical distribution it was scored as Afrotropical. Madagascar was treated as a separate area from the Afrotropical region.

We used three methods to reconstruct the ancestral distribution of hypothetical ancestors (internal nodes). Bayes-DIVA by Nylander et al. [52], alternative Bayes-DIVA by Harris and Xiang [58] implemented in S-DIVA (Statistical Dispersal-Vicariance Analysis) [59] and Dispersal-Extinction-Cladogenesis (DEC) in LaGrange [60, 61].

Both DIVA methods account for phylogenetic uncertainties by integrating over a sample of trees and represent improvements to DIVA, which was developed by Ronquist [62]. The original DIVA [62] is a parsimony based method which reconstructs the ancestral distribution of a group of organisms using a cost benefit metric in which dispersal event cost and vicariance events are free. DIVA reconstructs the ancestral area of a lineage without relying on any previous knowledge of the history of the areas (i.e. unlike cladistics biogeography) [52]. However, a practical drawback of the original DIVA method is that it can only handle single fully bifurcating trees, in effect ignoring phylogenetic uncertainty in the biogeographical reconstruction. This means that even weakly supported nodes in a phylogenetic tree are treated as true and without error which can mislead the ancestral history reconstruction [52, 63]. To get around this drawback, Nylander et al. [52] and Harris and Xiang [58] proposed Bayesian approaches that accounts for phylogenetic uncertainty and calculate probabilities for alternative biogeographic histories of lineages. Both methods apply the original DIVA algorithm to every tree in a pool of trees sampled from the posteriors distribution of topologies. The two Bayes-DIVA methods differ in the way they book-keep the frequency of multiple ancestral areas at a node F(r _y_)_t_ for each tree. F(r _y_)_t_ is the occurrence of ancestral range r at node y in tree t. Nylander et al. [52] record this fraction as 1/N, where N is the total number of alternative ancestral distributions at the node. This does not take into account the frequency of ancestral range r in all equally parsimonious pathways optimized by DIVA. Harris and Xiang [58] instead used the actual frequencies, i/R_t_, where i is the number of times range r occurs in the total number of MP pathways (R_t_) as optimized by DIVA.

Here we run both DIVA methods on the sample of 15000 trees of Hydaticini and Cybistrini datasets obtained from MrBayes with maximum areas at each node set to 2 [62]. As in the current S-DIVA version 1.9, the alternative-Bayes DIVA method is limited by the number of taxa and we therefore removed the outgroups and all but one representative from subgenus *Hydaticus*. All Madagascan species belong to subgenus *Prodaticus* and the monophyly of each is strongly supported [34] why we believe this does not affect the result other than potentially at the root node.

As both Bayesian DIVA methods are based on parsimony optimizations on each individual tree we also ran a model-based biogeographical analysis. Dispersal-Extinction-Cladogenesis (DEC) model described by Ree et al. [61] and Ree and Smith [60] is based on a stochastic model, in which evolution of geographic ranges both along a branch and at the node are modelled explicitly. It attempts to estimate how ancestral areas were inherited by daughter lineages. This model is evaluated in a likelihood framework, and the ancestral range inheritance scenario with the best likelihood of observing the current species distributions is chosen as optimal. We ran the DEC model with an equal constraints dispersal matrix in the software LaGrange [61] on the dated trees of Hydaticini and Cybistrini obtained from BEAST. To summarize the output from LaGrange we ran the BioGeoBEARS package [64] in R (www.r-project.org), to visualize the most probable ancestral distribution scenarios on tree nodes.

## Results

The combined datasets comprised 3011 characters for Hydaticini (2975 bp + 36 morphological characters) and 2800 characters for Cybistrini (2753bp +47 morphological characters). The attempt to sequence DNA from >100 year old dry-pinned museum material gave one 238 bp COI segment for *C*. *dytiscoides*, but was not successful for *H*. *plagiatus*. *Cybister dytiscoides* was included only in a single-gene COI analysis of Cybistrini to infer its approximate position (S1 Fig.), but not in the combined analysis due to the problems associated with missing data. In the COI tree *C*. *dytiscoides* came out in the subgenus *Cybister* clade as expected but in an unresolved position (S1 Fig.).

**Fig 2:**
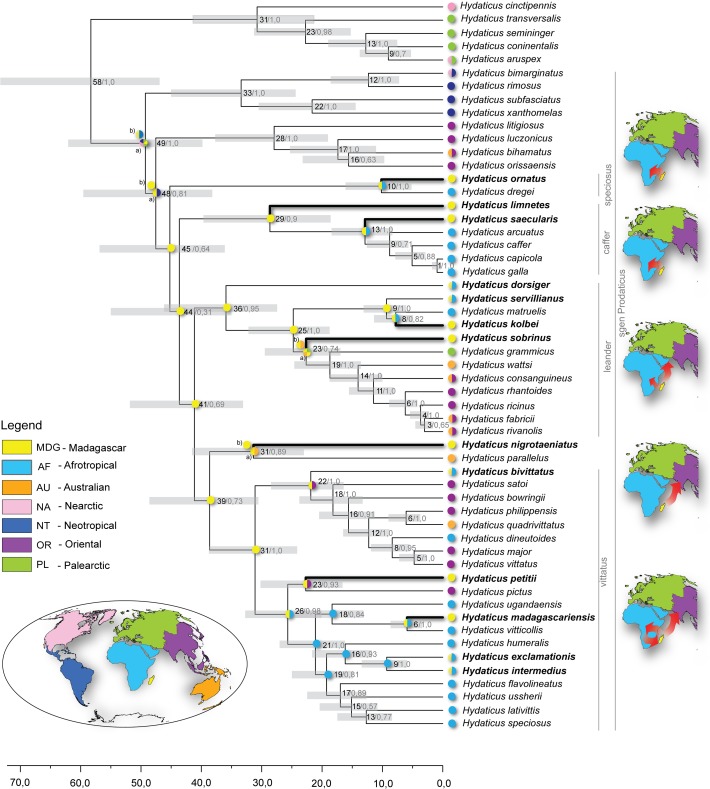
Timetree of Hydaticini. Time-calibrated Bayesian phylogeny obtained with BEAST. The numbers indicate estimated divergence time for each node/posterior probability support for the node. Maps represent zoogeographical areas used to obtain the ancestral distributions. Pie charts at internal nodes: a) Ancestral reconstruction obtained from the S-Diva methods, b) ancestral reconstruction obtained from the DEC method.

**Fig 3:**
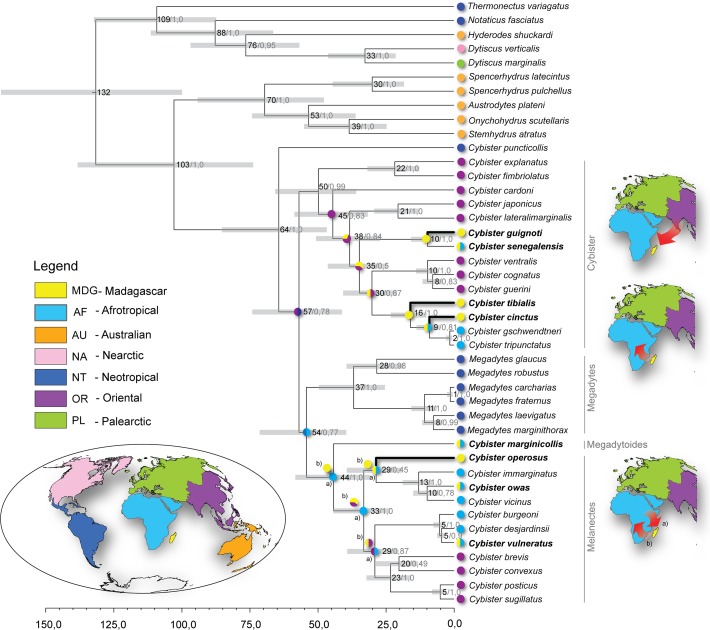
Timetree of Cybistrini. Time-calibrated Bayesian phylogeny obtained with BEAST. The numbers indicate estimated divergence time for each node/posterior probability support for the node. Maps represent zoogeographical areas used to obtain the ancestral distributions. Pie charts at internal nodes: a) Ancestral reconstruction obtained from the S-Diva methods, b) ancestral reconstruction obtained from the DEC method.

Species-group relationships were largely similar to those of Miller et al. [34, 36] and here we only focus on the phylogenetic affinity, age and origin of the added Malagasy species. One exception was that in the Bayesian Cybistrini analysis, Neotropical *Megadytes* was nested inside *Cybister* rendering the latter paraphyletic which was not recovered by Miller et al. [36]. This was, however, poorly supported and the ML analyses, instead, had *Megadytes* as sister to *Cybister*, also with low support (S2 Fig.). ML and Bayesian analyses, otherwise, obtained similar placements of Madagascar taxa, but with bootstrap values, in general, lower than posterior probability values (Figs. 2 and 3, S2 and S3 Figs.). Below we present only the result from the Bayesian analysis as the downstream biogeographic analyses accommodated phylogenetic uncertainty in a Bayesian context.

### Phylogenetic relationships

The phylogenetic analysis of Hydaticini placed the 13 species on Madagascar mostly with high support in different species groups, but the basal resolution between these species groups was not well supported (Fig. 2). The two endemic species *H*. *sobrinus* and *H*. *kolbei* were both recovered in the *leander* species group, but not as sister species. The two endemic *vittatus*-group species, *H*. *petitii* and *H*. *madagascariensis*, were neither recovered as sister taxa. *H*. *petitii* came out as basalmost in the *vittatus* species group together with oriental *H*. *pictus* with moderate support (pp = 0.93). *H*. *pictus* was prior to Miller et al [36] classified in its own genus. *H*. *madagascariensis* was instead nested deeper in the *vittatus* group and sister to *H*. *vitticollis* (pp = 1.0). *H*. *ornatus* was found as a sister species to the Afrotropical *H*. *dregei* in the *speciosus* group with strong support (pp = 1.0). The endemic *H*. *limnetes* and *H*. *saecularis* were placed with moderate (pp = 0.91) and high (pp = 1.0) support respectively as basal to the *caffer* species group but not as sister taxa. Endemic *H*. *nigrotaeniatus* came out as sister species to Australian *H*. *parallelus*, the relationship shown by Miller et al. [34] to be affected by long branch attraction. None of the non-endemic species on Madagascar came out as sister to any of the endemic species, but two of the non-endemic species, *H*. *exclamtionis* and *H*. *intermedius* were sisters (pp = 1.0).

The dataset of Cybistrini gave a phylogeny with mostly high support with regard to the placement of Malagasy species except for *C*. *operosus* (Fig. 3). Similar to Hydaticini, none of the endemic species of Cybistrini came out as sister species or monophyletic groups. Western Madagascan *C*. *tibialis* was recovered as basal to the *tripunctatus* group in which also the endemic *C*. *cinctus* belong. The endemic *C*. *operosus* with the, for the genus, unusual preference of occurring in running water, was recovered within the subgenus *Melanectes* (pp = 1.0), but without any close relatives. Endemic *C*. *guignoti* formed the sister species to non-endemic *C*. *senegalensis* (pp = 1.0). The remaining non-endemic subgenus *Melanectes* species *C*. *owas* and *C*. *vulneratus* formed separate rather recent colonizations. The monotypic subgenus *Megadytoides* with the widespread non-endemic *C*. *marginicollis* came out as basal to the subgenus *Melanectes* clade with strong support (pp = 1.0).

### Biogeographic analyses

All three biogeography methods produced highly similar results. The DEC model, based on a likelihood approach, indicated similar biogeographic scenarios as both S-DIVA methods, but with a few disagreements. Only for the estimated ancestral ranges of the nodes close to the root did Bayes-DIVA and alternative Bayes-DIVA show any differences.

For the Hydaticini phylogeny both DIVA and DEC approaches inferred Madagascar as the most likely ancestral range for the basal nodes in subgenus *Prodaticus* except the basalmost nodes connecting the Neotropical *subfasciatus* group and the Pacific/Oriental *pacificus* group. This unexpectedly suggests an out-of Madagascar scenario for the *vittatus*, *caffer*, *speciosus* and *leander* specious groups. The direction of out-of Madagascar dispersals pointed to both the Afrotropical and the Oriental region, somewhat more frequent to the former (S4, S5, and S6 Figs.).

The S-DIVA and DEC analyses for the Cybistrini dataset obtained similar ancestral areas over the tree except for the basal nodes of *Megadytoides*/*Melanectes*. The subgenus *Cybister* was inferred to come from an ancestor with an oriental distribution range with all methods (Fig. 3). Within this group there were either one or two dispersal events to Madagascar. For the origin of the *Megadytoides* + *Melanectes* the DEC model suggested the ancestor originated in Madagascar and dispersed out-of-Madagascar whereas S-DIVA suggested colonizations from Africa to Madagascar and also further to Oriental region within *Melanectes*. Consequently, the direction of dispersal between Africa and Madagascar for the non-endemic *C*. *owas* and *C*. *vulneratus* also differed between S-DIVA (Africa to Madagascar) and the DEC model (Madagascar to Africa). However, in the latter case the African origin was suggested as the second most probable scenario (S7, S8, and S9 Figs.).

### Dating

In Hydaticini the dating analysis estimated the crowngroup age of subgenus *Prodaticus* to 49 Ma (95%HPD: 40–62 Ma). As the biogeographic analysis inferred a Madagascan colonization already for the ancestor of the *vittatus*, *leander*, *caffer* and *speciosus* groups this was inferred to have occurred sometime between 45–49 Ma (HPD: 36–62 Ma). The multiple inferred out-of-Madagascar dispersal events in all four species groups were estimated to have occurred in the Oligocene and first half of Miocene. The only unambiguous dispersal event to Madagascar from Africa for the ancestor of endemic *H*. *madagascariensis* was inferred to be of Miocene origin 6–18 Ma (HPD: 3–24 Ma).

The dating analysis of Cybistrini estimated the crowngroup age of *Cybister* (incl. *Megadytes*) to 64 Ma (HPD: 47–85 Ma). In subgenus *Cybister* the single oriental-to-Madagascar colonization scenario was dated to around 38–35 Ma (HPD: 25–51 Ma). The scenario of two separate oriental-to-Madagascar colonization events would have occurred sometime between 10–35 Ma (HPD: 5–46 Ma). *Cybister operosus* of the *Melanectes* clade seem to be the oldest endemic lineage in the tribe. Under the S-DIVA optimizations the ancestor of *C*. *operosus* colonized Madagascar from Africa 29–33 Ma (HPD: 20–44 Ma).

## Discussion

Many endemic species of Madagascar seem to originate from one or only a few transoceanic dispersal events to the island, which subsequently gave rise to highly diverse groups. For example, lemurs on Madagascar can be traced back to a single colonization event about 49–66 million years ago but have diverged into more than 100 endemic species [1, 4, 12]. A Similar pattern of single or just a few colonization events has also been found in various insect groups. For example, Allodopine bees on Madagascar seem to originate from two colonization events which have resulted in 35 endemic taxa [20, 21]. Fungus-growing termites of Madagascar all originate from a single colonization event [24]. The mayfly genus *Compsoneuria* in Madagascar was inferred to have a single origin with a lineage in mainland Africa as sister [65]. Canthonini dung beetles were estimated to have colonized Madagascar in a single event and later evolved into multiple endemic taxa [18]. But there are also examples of multiple colonization events without significant species radiations. The analysis of pierid butterflies suggests that major diversification in *Colotis* occurred in Africa with at least seven subsequent dispersals to Madagascar producing eight endemic species [22]. It has been reported that *Nephila* spiders colonized Madagascar more than ten times without any species radiation [6, 66]. The large-bodied diving beetles in the tribes Hydaticini and Cybistrini, analyzed here, seems to follow this second pattern. The history of Cybistrini at least can be summarized as multiple colonizations with no species radiations. Similarly, the analysis of Hydaticini indicated that the 13 species on Madagascar did not form any monophyletic groups or even a single example of a sister-group relationship between two endemic species.

The lack of endemic clades within this group can have different explanations. Firstly, for some reason, biotic and or abiotic factors related to the island, the organisms, or some interaction did not provide favorable conditions for *in situ* radiations. Second, there might have been *in situ* radiations but within which descendants also spread to other regions rendering such radiations paraphyletic. Lastly, there might have been *in situ* radiations but past, or even recent anthropogenically mediated extinctions, might have left but a fraction of the original diversity in the group.

Concerning the first option, there have been several studies focusing on hypotheses explaining what environmental factors could serve as barriers for gene flow on Madagascar leading to speciation. Wilme et al. [28] proposed that areas between river catchment systems at low elevations, were zones of isolation during periods of drier climate and hence led to speciation of locally endemic species, especially vertebrates. Raxworthy et al. [67], on the other hand, highlighted the climatic gradients across Madagascar and suggested these might have been the driving force behind the microendemic pattern seen in many groups today. Pearson and Raxworthy [68] tested to what degree distribution data of lemurs, and three groups of reptiles, coincided with the predicted areas of endemism from each hypothesis. They found support for both the watershed and the climate hypothesis in different terrestrial vertebrates. However, most insects are capable of flight and the larger rivers on the island are unlikely to constitute dispersal barriers, at least for insect with the flight capacity similar to most large diving-beetles. Insects can also be transferred by wind or water currents long distances. Our results suggest that neither the parameters in the “watershed hypothesis” nor of the ”current climate hypothesis” have been significant gene flow barriers for species of Hydaticini and Cybistrini.

For the Hydaticini clade, our results from the biogeographical analysis instead strongly favored the second, out-of-Madagascar option. According to this analysis most of the endemic species do stem from a number of rather old *in situ* speciation events, but where descendants later spread to the African or Oriental regions. Out-of-Madagascar dispersal events have been inferred for a number of other groups as well. Especially, the surrounding Mascarene and Comoros islands have been the recipients of flora and fauna from Madagascar [7, 69–74] but also a number of cases to mainland Africa [71, 72, 75, 76]. Similar “out of island” dispersal patterns were inferred also from other insular biodiversity hotspots. Balke et al., [77, 78] recognized the island of New Guinea as a “biodiversity pump” from which Colymbetinae *Rhantus* diving beetles dispersed both to Australia and jumped across Wallace's line to Oriental and Palearctic regions [79]. Filardi and Moyle [80] called such dispersal “upstream colonization”, and exemplified it with a pan-Pacific bird group which recolonized continental areas from remote islands. Another example is the Hawaiian diversification and subsequent out-of-Hawaii dispersal of Drosophilid flies [81].

Sanmartin and Ronquist [82] evaluated the frequency of dispersal and showed that the dispersal from Madagascar to Africa was higher than from Africa to Madagascar sometime during Cretaceous and early-Tertiary. Our estimated divergence times of endemic species are younger; around Oligocene and Miocene so they do not fit this pattern even though the age estimates should be taken with great caution (see discussion below). Moreover, most out-of-Madagascar examples have been of clades where the majority of the global diversity also occurs on Madagascar. Madagascan origins are in those cases also supported by the common correlation between clade age and diversity [83, 84]. The present analysis of Hydaticini seems to suggest a Madagascan origin for a clade most diverse in the Afrotropical and Oriental regions, and only a minority of species in Madagascar. We are not aware of any previous examples of this, and while being a very bold and intriguing hypothesis, we also present a potential caveat related to differences in sampling intensity in natural habitats (see below).

Independent of the direction, the timing of these transoceanic dispersal events is in agreement with a general pattern seen in other groups as reviewed by Yoder and Novak [1]. Most endemic taxa of Madagascar can be traced to a most recent common ancestor in the Cenozoic. Madagascar was for the entire Cenozoic period an island surrounded by ocean, hence transoceanic dispersal is the sole option for this time period. Previous recurs to Gondwana break-up vicariance explanations have largely been abandoned since the age of phylogenetic dating. A recent study by Samonds et al. [85] focused on dispersal abilities and rates of arrival over time for Malagasy vertebrate groups by classifying them into freshwater obligate, terrestrial and dispersal-advantaged. They concluded that during the Late Jurassic, when eastern Madagascar was still attached to India and its south was flanked by Antarctica/Australia, the rate of arrival was highest for freshwater ancestors. In contrast, during the Cenozoic the arrival rate for dispersal-advantaged taxa was three times higher than that of dispersal-disadvantaged taxa. It has been reported that adult diving beetles in general have good flight capacity which would place them in the dispersal-advantaged category, but for most species it has never actually been quantified. Witness to their dispersal capacity, however, can be seen in for example the colonization of remote volcanic islands like Galapagos [86], Hawaii [87], Tristan da Cunha and Robinson Crusoe Island, Juan Fernández [88]. The distance between the African mainland and Madagascar, across the Mozambique Channel, is 430 km and has remained fairly constant during the Cenozoic. Jackson [35] discussed the hypothesis that water beetles may possibly be carried by air currents (e.g. storms), which would allow them to be transported long distances.

Ali and Huber [27] suggested that during the Paleogene period strong sporadic surface currents occurred, e.g. due to storms, from northeast Mozambique and Tanzania eastward towards Madagascar. Moreover, the authors indicated that by early Miocene the currents were perennially directed towards Africa, making dispersal from Africa much more difficult. This is in agreement with our findings that the colonization from Madagascar to Africa occurred in most Malagasy lineages, sometime around Miocene. As diving beetles pupate in the soil next to waterbodies the scenario of rafting on mats of vegetation broken off and washed out to the sea by rivers is also a plausible means of transportation. Although these beetles are aquatic, and some species even adapted to salinity levels equal to or greater than seawater [89], no diving beetles can persist in open ocean or even in truly marine conditions. Salinity might not be the greatest obstacle for an open ocean journey and we find flying or rafting more likely than swimming when it comes to Cenozoic colonizations of Madagascar.

However, phylogenetic dating analyses inevitably come with a large degree of uncertainty [90]. Clock model choices aside, the treatment of external calibration data in the analyses is probably the most critical step in the treatment of uncertainty. Treating a known fossil as a point age of a node will bias the result, but this was mostly a shortcoming of older methods not being able to handle prior distributions or minimum age constraints [91]. Incorrectly placed external information, e.g. a fossil record, within the tree may also bias the results, and severely so. The recent study by Bukontaite et al. [42] showed this uncertainty. Whether a fossil was used as a stem- or crowngroup constraint of a genus resulted in significantly different age estimates in the tribe Aciliini, with one being in accordance with a Gondwana break-up origin and the other not. Moreover, use of a single fossil calibration point might generate unpredictable and variable divergence dates and, if available, multiple fossil calibrations points are always preferable [92]. However, the reality is far from ideal and for many groups of organisms the fossil record is exceedingly sparse, and in order to do any kind of calibration, one will have to do with a single suboptimal calibration point [91]. Alternatively, geological events can be used to date trees if fossils are unreliable, or unknown, as we did here for Cybistrini. Also biogeographical based calibrations might over- or underestimate the divergence times if a lineage split have appeared long before or after the geological event [93, 94]. There are also considerable error margins around the geologically estimated dates of separation between continental areas or volcanic islands. For example, the estimated timing of the India and Madagascar separation vary between 160 to 80 Mya [5 and reference therein, 60]. The latter estimate, or close to it, is preferred by most but it is often unknown how long the final separation of continents took [93]. For Cybistrini we used the geological age estimate of the volcanic island Mauritius but very different geological age estimates exist depending on what strata of the island is dated [73, 95, 96]. More generally using ages of islands as maximum age constraints of island endemics is potentially problematic [96]. Most island systems of volcanic origin represent but the tip of the iceberg of much larger seamount provinces [97, 98]. Seamounts which are eroded down below the surface today may at various times in the past have been above surface providing habitat for terrestrial lineages [99]. Consequently, endemic island taxa may have evolved on neighboring older islands which are today submerged seamounts and using an age constraint based on present islands may be too conservative [96]. Mauritius is situated at the southern end of the Mascarene plateau which extends north to the Seychelles Plateau of continental Gondwanan origin. Age estimates at drilling sites along the Mascarene plateau support a hotspot volcanic origin with younger sites in the south and older towards the north [100]. The mean age estimates of nodes in our analyses should therefore be taken with caution, but the general pattern of multiple Cenozoic transoceanic dispersals, whether by flight, rafting or drifting, should be a stable conclusion nevertheless. Also the Mauritius taxa used, *Cybister desjardinsii*, is very similar both genetically and morphologically to the widespread species *C*. *vulneratus* and the assumption used does at least not seem way off. If we use the higher level dating analysis on Coleoptera [101] based on multiple fossils to calibrate the utrametric family tree of Dytiscidae by Ribera et al [102], we receive an approximate date on stem/crown group Cybistrini at 140/85 Ma and Hydaticini at 95/55 Ma. This is reasonably similar to the present analyses giving Cybistrini stem/crown at 132/103 Ma and Hydaticini at 79/58 Ma. The larger difference for Cybistrini could stem from the placement of Cybistrini in Ribera et al's analysis [102] which is not supported in later analysis [103]. An inappropriate endemic island taxa constraint sensus Heads [96] would result in over conservative estimates, biased towards younger ages, which does not seem to be the case. Likewise we can see from the estimated crown age of the genus *Cybister* (including *Megadytes*) from both our analysis (64Ma) and Ribera et al's [102] as scaled above (65 Ma) that the known *Cybister* fossil [33] from the Miocene period ending 5.3 Mya are too recent fossil to be useful for calibration unless cladistics analysis can place them with higher precision in the genus.

The methods Bayes-DIVA and alternative Bayes-DIVA, which apply DIVA on a sample of trees, can give very different results [58]. The method suggested by Nylander et al. [52] weights all alternative ancestral distribution ranges at a node as equal fractions. Alternatively Bayes-DIVA [58] instead uses the real range frequencies from the pool of all equally parsimonious pathways optimized by DIVA, and this was found by Harris and Xiang to sometimes make a large difference. However, in both methods, inferred ancestral areas can become spurious if there have been extinctions, local speciation, or the formation of widespread species within the group [52, 51]. The maximum likelihood model (DEC) has similar assumptions to those of DIVA (see Fig. 4 in [104]), except that DEC allows peripatric speciation, which results in one descendant occupying the area of speciation whereas the other inherits the entire range and, unlike DIVA, it does not permit vicariance scenario in which each daughter occupies more than one area. Also, ancestral areas are reconstructed over a single topology and it does not account for phylogenetic uncertainty [60, 61]. Despite differences in the underlying biogeographical model, DEC and S-DIVA often agree on the ancestral distributions (e.g [105, 106]. We observed similar consistency in all three models of our analyses. Only in a few cases did the models estimate different ancestral areas for a node. In particular, all three estimated an early origin for *Hydaticus* in Madagascar and multiple out-of-Madagascar dispersal events instead of multiple independent colonization events. This was due to the endemic Madagascar species being widely spread throughout the phylogeny and having well-supported basal placements in several species groups. Hence, the unusually bold hypothesis of a Madagascar origin for this group, which is most diverse in Africa and the Orient, cannot be discarded as an artefact from the assumptions of a single method. Nevertheless, a challenger to this hypothesis could be sought in another sort of methodological artefact. The inclusion of the near complete known fauna for Madagascar of this group was achieved after intense sampling across all of Madagascar over the last eight years. Several of the endemic lineages occupying basal positions in species groups were very rare and found once or twice and always in natural, unspoiled habitats. The mainland African continent has simply not been sampled as intensely and the majority of the included African species perhaps being the more widespread fauna. We suspect that a similarly intense sampling on mainland Africa, in particular in unspoiled natural habitats, will reveal new species challenging the Madagascan dominance at basal positions across species groups, which may turn the pendulum in the biogeographic analysis.

Finally, the third potential explanation, as mentioned above, relates to past and present extinctions, which may affect inferred patterns. Endemic freshwater organisms on Madagascar are severely threatened by human activities in Madagascar [32]. Slash and burn agriculture, in Madagascar known as 'tavy', eutrophication by zebu cattle and introduction of commensal fish, are all imminent threats to the few remaining natural environments and the endemic freshwater fauna. However, conservation efforts are entirely dependent on improved political and economic situations in the country. We were not able to find two of the described endemic species of Cybistrini and Hydaticini despite extensive field work, suggesting these species might already be extinct. Several other species are exceedingly rare and threatened, like the lowland *H*. *madagascariensis*, and the western deciduous forest inhabiting *C*. *tibialis*. Each loss of an endemic species in this group represent the irreversible loss of a 10–40 Ma evolutionary lineage trajectory that started with a strenuous transoceanic dispersal event, the nature of which we are only starting to understand.

## Conclusions

In this study we tested the evolutionary origin of the Malagasy representatives of the tribes Hydaticini and Cybistrini. There was a conspicuous lack of endemic species forming sister-group relationships or monophyletic groups in both tribes. We speculate that the lack of species radiations for this group in Madagascar, is due to their dispersal ability where neither the size of the island, larger rivers, nor climatic gradients represent significant barriers to gene flow. All interactions with surrounding continents were dated to be of Cenozoic origin, between 40 Ma and the present, and therefore must represent transoceanic dispersals since Madagascar has been an isolated island for about 90 Ma. The biogeographic analyses, independent of model, inferred for Hydaticini an early Madagascan colonization followed by multiple out-of-Madagascar dispersals, but the robustness to this hypothesis remain to be tested with an equally intense sampling in natural habitats on the African mainland as on Madagascar.

## Supporting Information

S1 Fig COI gene tree with *Cybister dytiscoides* included based on a short fragment. (PDF)

S2 Fig Phylogenetic tree of Hydaticini based on ML analysis with bootstrapping. (PDF)

S3 Fig Phylogenetic tree of Cybistrini based on ML analysis with bootstrapping. (PDF)

S4 Fig Ancestral distribution reconstruction of Hydaticini using Bayes-DIVA by Nylander et al., (2008). (PDF)

S5 Fig Ancestral distribution reconstruction of Hydaticini using Bayes-DIVA by Harris and Xiang (2009). (PDF)

S6 Fig Ancestral distribution reconstruction of Hydaticini using the DEC approach. (PDF)

S7 Fig Ancestral distribution reconstruction of Cybistrini using Bayes-DIVA by Nylander et al., (2008). (PDF)

S8 Fig Ancestral distribution reconstruction of Cybistrini using Bayes-DIVA by Harris and Xiang (2009). (PDF)

S9 Fig Ancestral distribution reconstruction of Cybistrini using the DEC approach. (PDF)

S1 Table Specimen data and genbank accession numbers. (PDF)
