# Supplementary figures and images for: In or Out-of-Madagascar?—Colonization Patterns for Large-Bodied Diving Beetles (Coleoptera: Dytiscidae)
Source: PLoS One. 2015 Mar 20;10(3):e0120777. doi: 10.1371/journal.pone.0120777 (PMC4368551; doi:10.1371/journal.pone.0120777)

Fig S1.

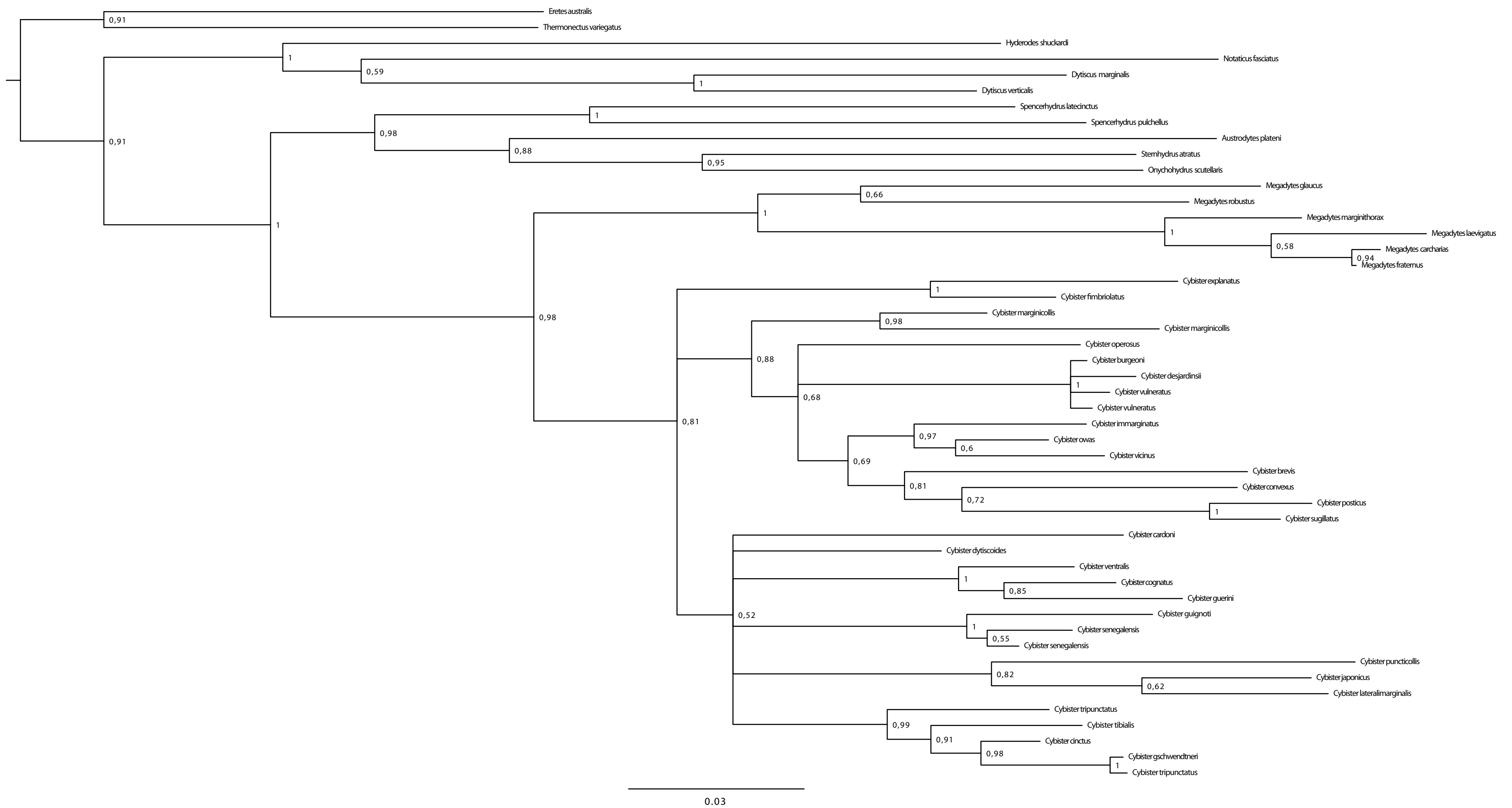

Supplement: S1 Fig — (PDF) [file pone.0120777.s001.pdf]

Fig S2.

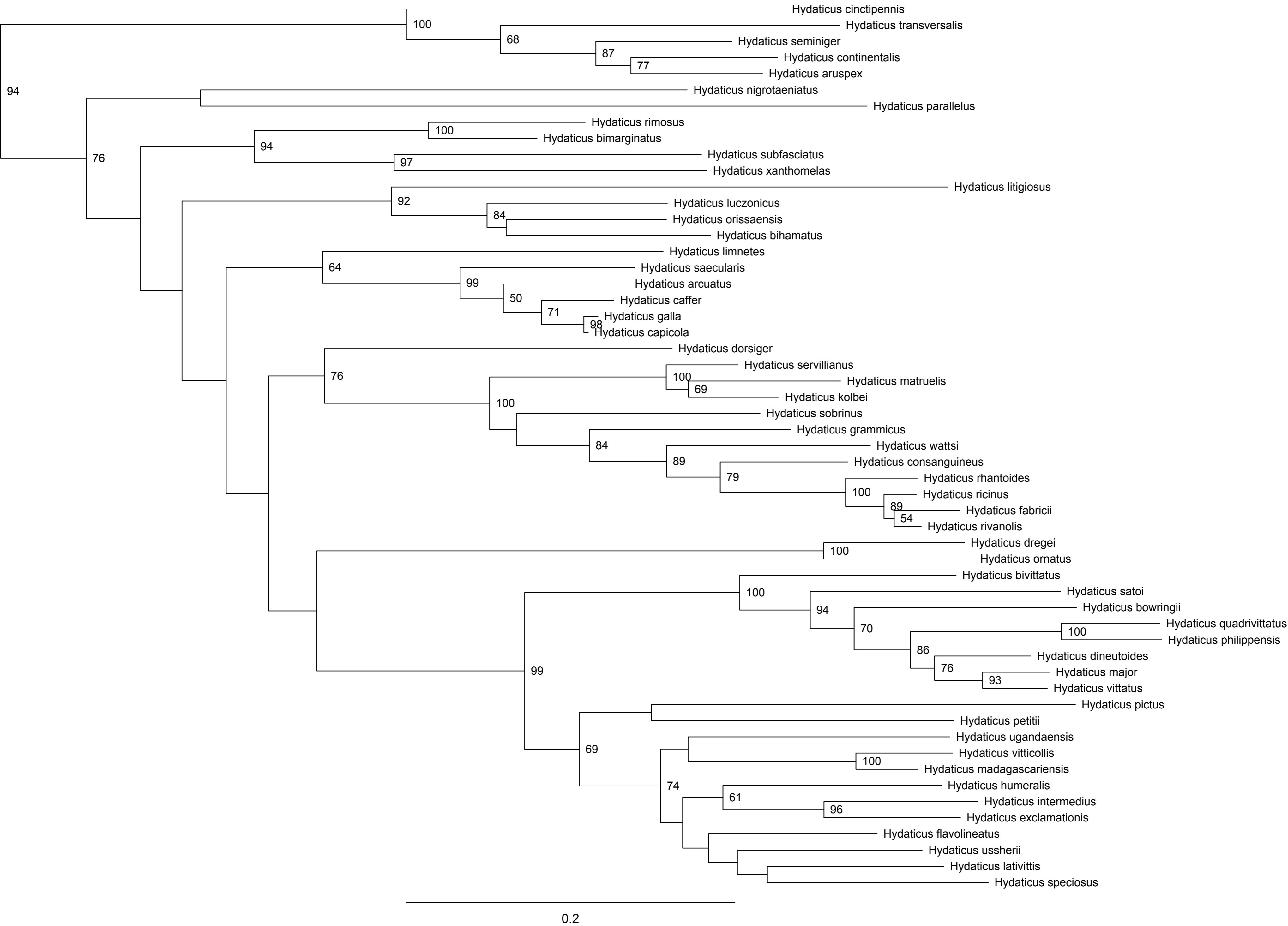

Supplement: S2 Fig — (PDF) [file pone.0120777.s002.pdf]

Fig S3.

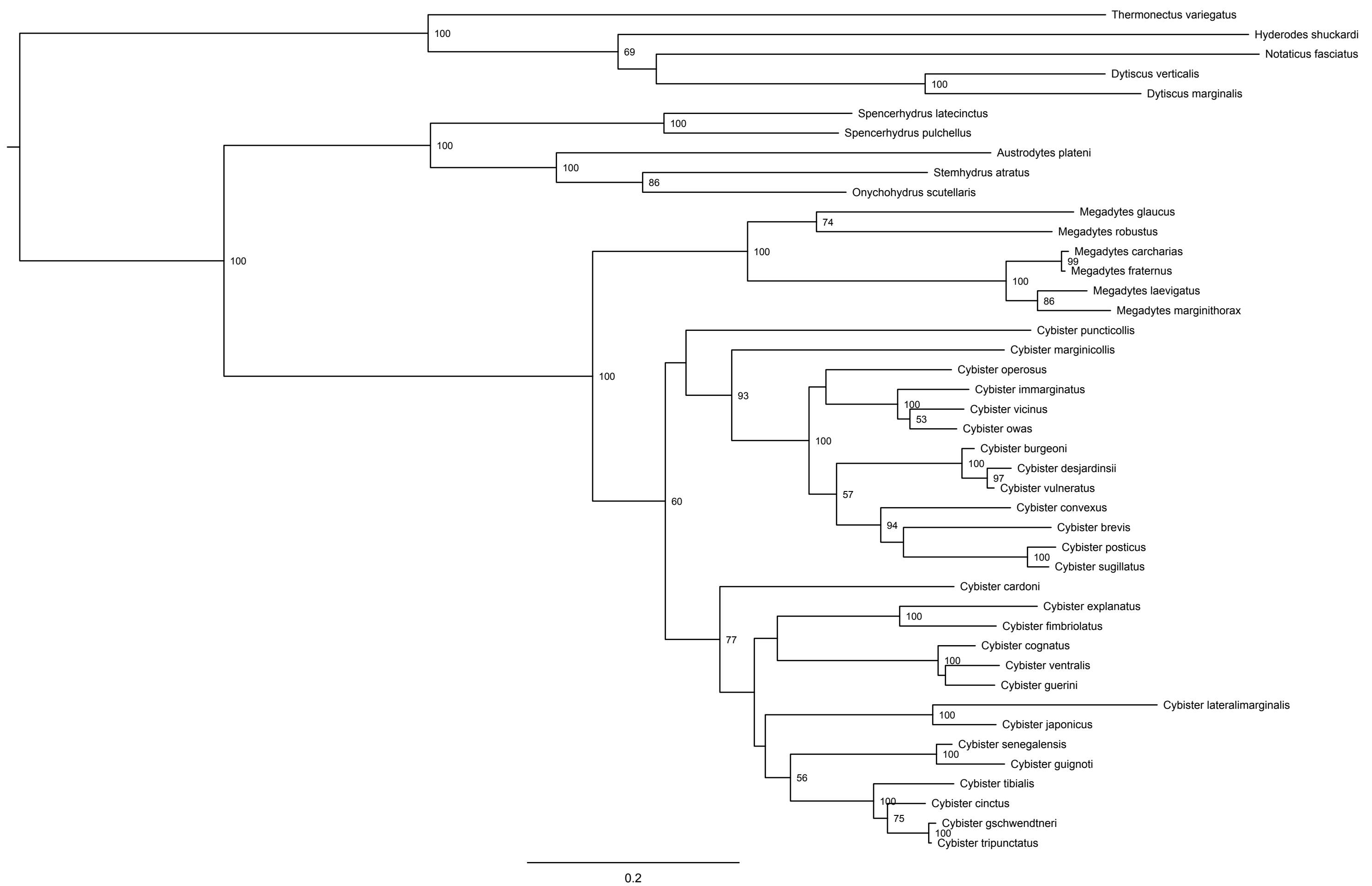

Supplement: S3 Fig — (PDF) [file pone.0120777.s003.pdf]

Fig S5.

## Bayes-DIVA by Harris and Xiang (2009)

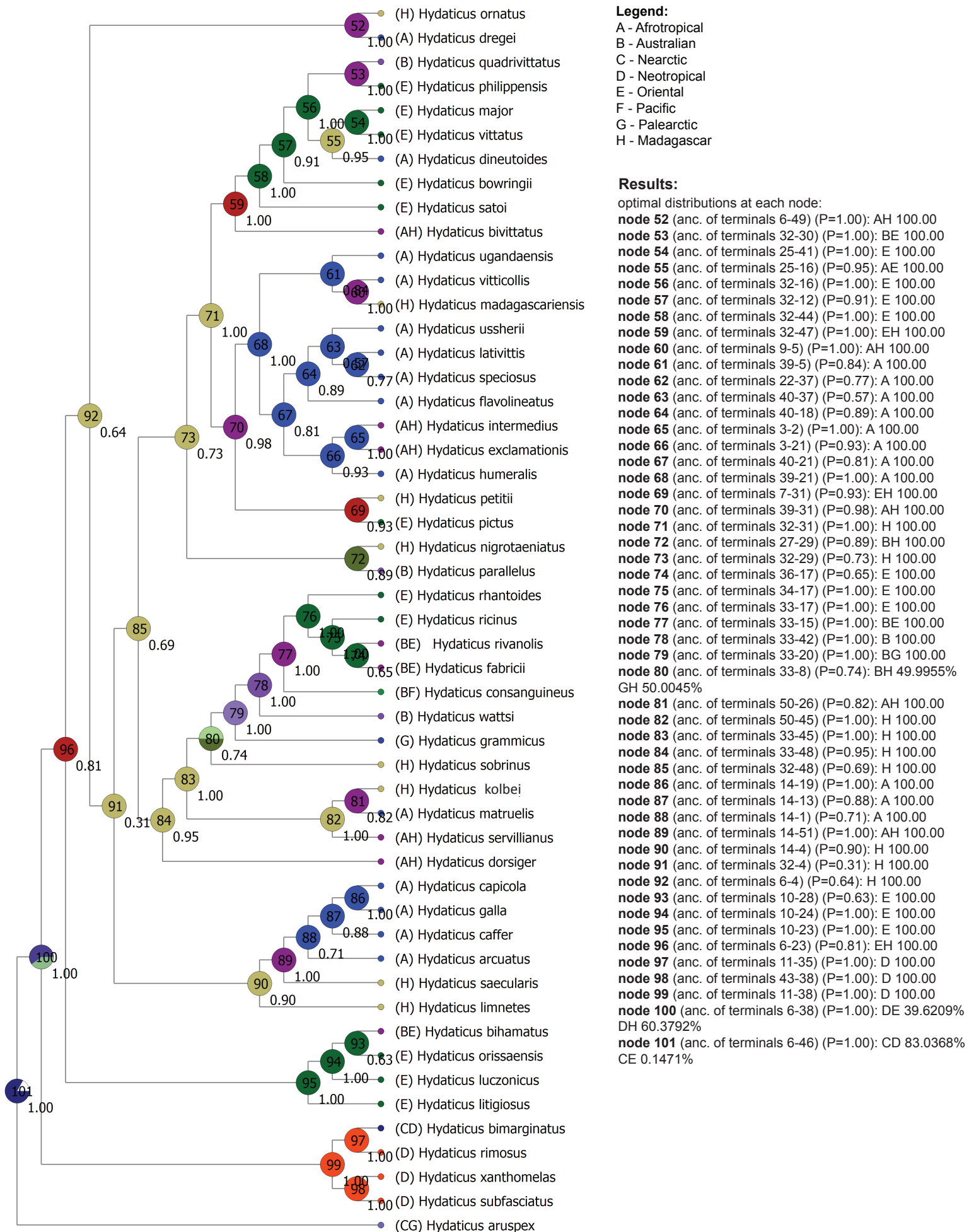

Supplement: S5 Fig — (PDF) [file pone.0120777.s005.pdf]
