# Supplementary material for: In or Out-of-Madagascar?—Colonization Patterns for Large-Bodied Diving Beetles (Coleoptera: Dytiscidae)
Source: PLoS One. 2015 Mar 20;10(3):e0120777. doi: 10.1371/journal.pone.0120777 (PMC4368551; doi:10.1371/journal.pone.0120777)
Supplement: S4 Fig — (PDF) [file pone.0120777.s004.pdf]

Fig S4.

Bayes-DIVA by Nylander et al. (2008)

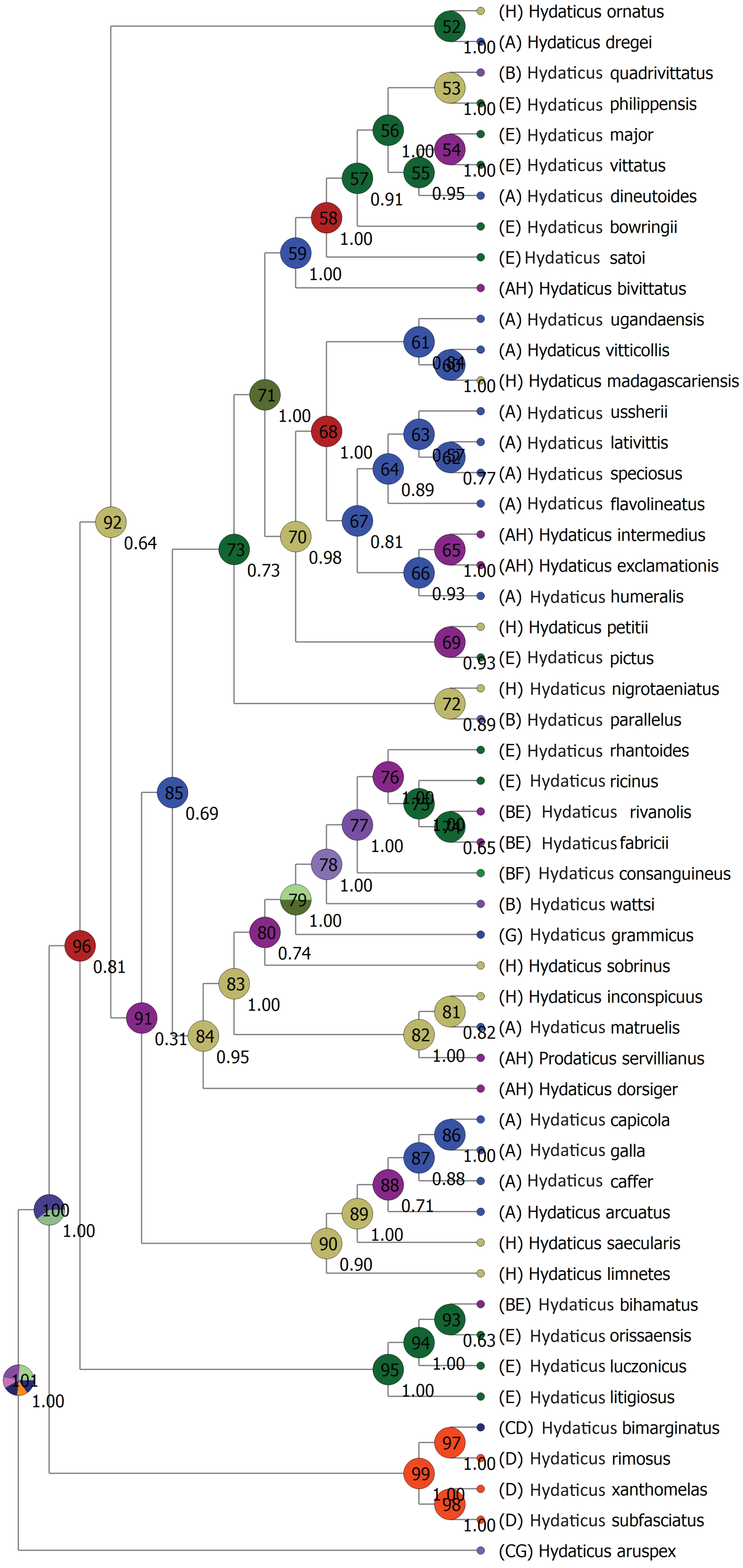

Legend:
A - Afrotropical
B - Australian
C - Nearctic
D - Neotropical
E - Oriental
F - Pacific
G - Palearctic
H - Madagascar

Results:

optimal distributions at each node:
node 52 (anc. of terminals 25-41) (P=1.00): E 100.00
node 53 (anc. of terminals 25-16) (P=0.95): AE 100.00
node 54 (anc. of terminals 30-32) (P=1.00): BE 100.00
node 55 (anc. of terminals 25-32) (P=1.00): E 100.00
node 56 (anc. of terminals 25-12) (P=0.91): E 100.00
node 57 (anc. of terminals 44-12) (P=1.00): E 100.00
node 58 (anc. of terminals 44-47) (P=1.00): EH 100.00
node 59 (anc. of terminals 3-2) (P=1.00): A 100.00
node 60 (anc. of terminals 3-21) (P=0.93): A 100.00
node 61 (anc. of terminals 22-37) (P=0.77): A 100.00
node 62 (anc. of terminals 22-40) (P=0.57): A 100.00
node 63 (anc. of terminals 22-18) (P=0.89): A 100.00
node 64 (anc. of terminals 3-18) (P=0.81): A 100.00
node 65 (anc. of terminals 9-5) (P=1.00): AH 100.00
node 66 (anc. of terminals 39-5) (P=0.84): A 100.00
node 67 (anc. of terminals 3-5) (P=1.00): A 100.00
node 68 (anc. of terminals 31-7) (P=0.93): EH 100.00
node 69 (anc. of terminals 3-7) (P=0.98): AH 100.00
node 70 (anc. of terminals 44-7) (P=1.00): H 100.00
node 71 (anc. of terminals 27-29) (P=0.89): BH 100.00
node 72 (anc. of terminals 44-29) (P=0.73): H 100.00
node 73 (anc. of terminals 17-36) (P=0.65): E 100.00
node 74 (anc. of terminals 34-36) (P=1.00): E 100.00
node 75 (anc. of terminals 34-33) (P=1.00): E 100.00
node 76 (anc. of terminals 34-15) (P=1.00): BE 100.00
node 77 (anc. of terminals 34-42) (P=1.00): B 100.00
node 78 (anc. of terminals 34-20) (P=1.00): BG 100.00
node 79 (anc. of terminals 34-8) (P=0.74): BH 50.00 GH 50.00
node 80 (anc. of terminals 26-50) (P=0.82): AH 100.00
node 81 (anc. of terminals 45-50) (P=1.00): H 100.00
node 82 (anc. of terminals 34-50) (P=1.00): H 100.00
node 83 (anc. of terminals 34-48) (P=0.95): H 100.00
node 84 (anc. of terminals 44-48) (P=0.69): H 100.00
node 85 (anc. of terminals 14-19) (P=1.00): A 100.00
node 86 (anc. of terminals 14-13) (P=0.88): A 100.00
node 87 (anc. of terminals 14-1) (P=0.71): A 100.00
node 88 (anc. of terminals 51-1) (P=1.00): AH 100.00
node 89 (anc. of terminals 4-1) (P=0.90): H 100.00
node 90 (anc. of terminals 44-1) (P=0.31): H 100.00
node 91 (anc. of terminals 49-6) (P=1.00): AH 100.00
node 92 (anc. of terminals 44-6) (P=0.64): H 100.00
node 93 (anc. of terminals 10-28) (P=0.63): E 100.00
node 94 (anc. of terminals 10-24) (P=1.00): E 100.00
node 95 (anc. of terminals 10-23) (P=1.00): E 100.00
node 96 (anc. of terminals 44-23) (P=0.81): EH 100.00
node 97 (anc. of terminals 43-38) (P=1.00): D 100.00
node 98 (anc. of terminals 11-35) (P=1.00): D 100.00
node 99 (anc. of terminals 43-35) (P=1.00): D 100.00
node 100 (anc. of terminals 44-35) (P=1.00): DE 39.6167 DH 60.3833
node 101 (anc. of terminals 44-46) (P=1.00): CD 15.3867 CE 10.9671 DG 15.3867 EG 10.9671 CH 23.6462 GH 23.6462
