# Supplementary material for: In or Out-of-Madagascar?—Colonization Patterns for Large-Bodied Diving Beetles (Coleoptera: Dytiscidae)
Source: PLoS One. 2015 Mar 20;10(3):e0120777. doi: 10.1371/journal.pone.0120777 (PMC4368551; doi:10.1371/journal.pone.0120777)
Supplement: S6 Fig — (PDF) [file pone.0120777.s006.pdf]

Fig S6.

DEC model (Ree et al., 2005 and 2008)

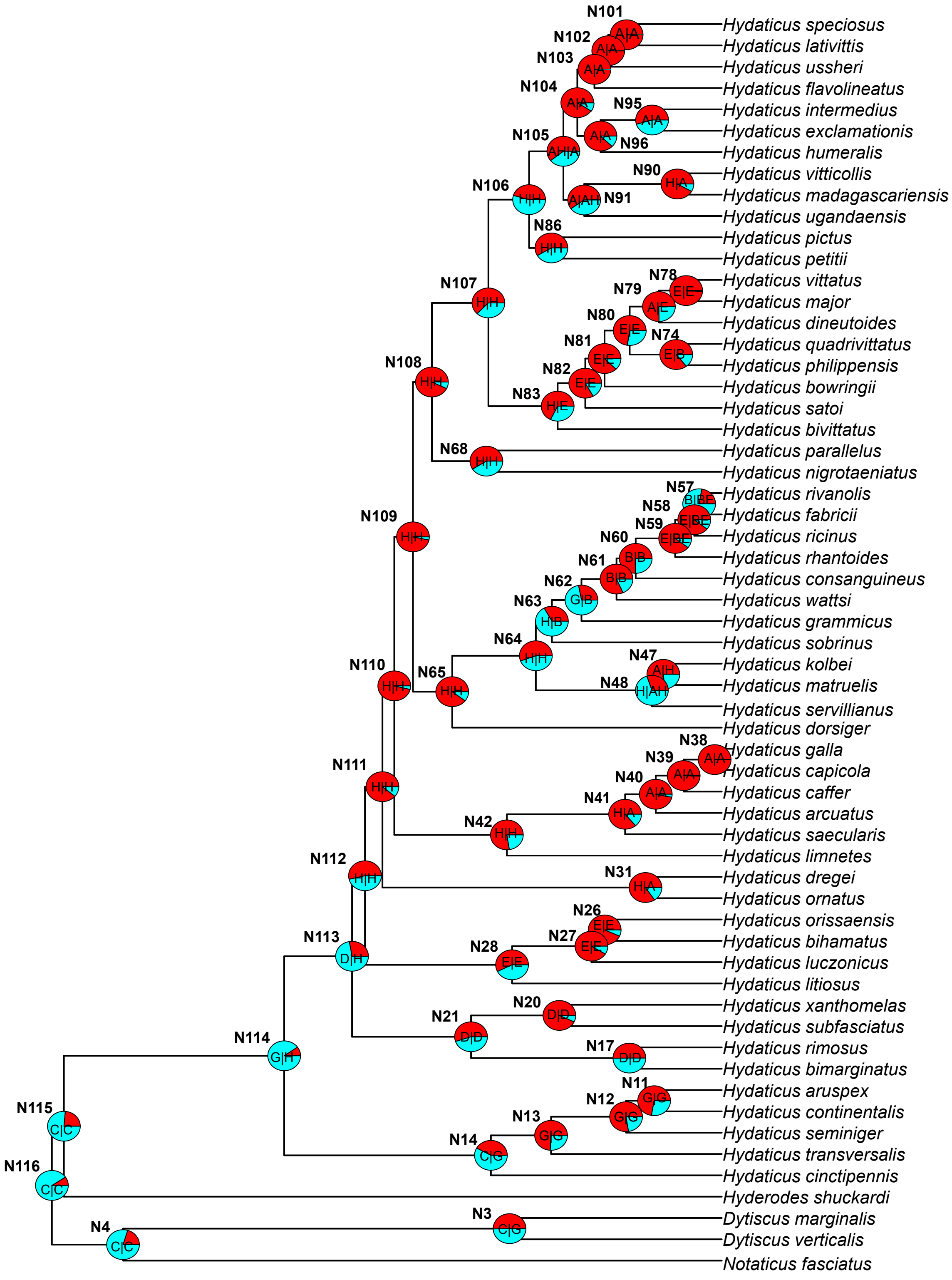

Legend:
A - Afrotropical
B - Australian
C - Nearctic
D - Neotropical
E - Oriental
F - Pacific
G - Palearctic
H - Madagascar

Results:

node N116: [C|C] 0.09431, [G|G] 0.065, [CD|C] 0.05444, [C|BC] 0.04781, [D|D] 0.04002
node N4: [C|C] 0.2042, [G|G] 0.1646, [D|D] 0.08331, [D|C] 0.06881, [D|G] 0.05778
node N3: [C|G] 0.5027, [C|CG] 0.1094, [CG|G] 0.1094, [C|C] 0.06733, [G|G] 0.05892
node N115: [C|C] 0.2366, [G|G] 0.1619, [D|D] 0.1345, [H|H] 0.06674, [B|C] 0.02856
node N114: [G|H] 0.09952, [C|C] 0.0993, [G|D] 0.09929, [C|H] 0.08826, [C|D] 0.08805
node N14: [C|G] 0.419, [C|CG] 0.1323, [CG|G] 0.1013, [G|G] 0.1006, [C|C] 0.09797
node N13: [G|G] 0.7423, [G|CG] 0.1828, [CG|G] .02726
node N12: [G|G] 0.7742, [G|CG] 0.2104
node N11: [G|G] 0.724, [G|CG] 0.2635
node N113: [D|H] 0.2772, [C|H] 0.1585, [H|H] 0.09856, [D|DH] 0.05197, [DH|H] 0.04723
node N21: [D|D] 0.5514, [CD|D] 0.2508, [DH|D] 0.04461, [D|DH] 0.04448, [C|D] 0.01776
node N17: [D|D] 0.5019, [CD|D] 0.4537
node N20: [D|D] 0.9291, [DH|D] 0.0128, [D|DH] 0.0128
node N112: [H|H] 0.5388, [E|H] 0.1656, [D|H] 0.06988, [C|H] 0.04777, [EH|H] 0.03582
node N28: [E|E] 0.5742, [EH|E] 0.1936, [E|EH] 0.09023, [DE|E] 0.03093, [CE|E] 0.02182
node N27: [E|E] 0.9114, [E|BE] 0.03464, [EH|E] 0.02527
node N26: [E|E] 0.932, [BE|E] 0.05415
node N111: [H|H] 0.8883, [AH|H] 0.03395, [EH|H] 0.0155, [DH|H] .0102, [H|EH] 0.00948
node N31: [H|A] 0.8441, [H|AH] 0.05487, [AH|A] 0.05487
node N110: [H|H] 0.9617
node N42: [H|H] 0.7747, [H|AH] 0.1844
node N41: [H|A] 0.8612, [AH|A] 0.07311, [H|AH] 0.03504
node N40: [A|A] 0.9683
node N39: [A|A] 0.9951
node N38: [A|A] 0.9996
node N109: [H|H] 0.9662
node N65: [H|H] 0.8952, [AH|H] 0.03969, [H|BH] 0.02022
node N64: [H|H] 0.5572, [H|BH] 0.2088, [H|GH] 0.0992, [AH|H] 0.089
node N48: [A|AH] 0.2996, [H|AH] 0.2996, [H|H] 0.2114, [AH|H] 0.08865, [AH|A] 0.08865
node N47: [A|H] 0.7668, [H|H] 0.1188, [AH|H] 0.03716, [A|AH] 0.03716
node N63: [H|B] 0.3289, [H|H] 0.3163, [H|G] 0.1679, [BH|B] 0.05435, [H|BH] 0.04637
node N62: [G|B] 0.279, [B|B] 0.2401, [H|B] 0.2157, [H|H] 0.04131, [BG|B] 0.0366
node N61: [B|B] 0.8156, [B|BE] 0.07116, [BH|B] 0.04269, [BG|B] 0.0224
node N60: [B|B] 0.7476, [B|BE] 0.1097, [B|E] 0.07316, [BF|B] 0.05351
node N59: [E|BE] 0.8949, [E|E] 0.08787
node N58: [E|BE] 0.8928, [E|E] 0.08731
node N57: [BE|E] 0.2253, [E|BE] 0.2253, [BE|B] 0.2253, [B|BE] 0.2253, [E|E] 0.07628
node N108: [H|H] 0.9276, [BH|H] 0.02633
node N68: [H|H] 0.5902, [H|B] 0.245, [H|BH] 0.06071, [BH|B] 0.06071
node N107: [H|H] 0.6286, [EH|H] 0.1001, [A|AH] 0.09657, [H|AH] 0.06159, [EH|E] 0.02855
node N83: [H|E] 0.6783, [A|E] 0.06817, [H|H] 0.04776, [EH|E] 0.04548, [AH|A] 0.04523
node N82: [E|E] 0.831, [E|AE] 0.08737, [EH|E] 0.0458
node N81: [E|E] 0.8732, [E|AE] 0.09832
node N80: [E|E] 0.7256, [E|AE] 0.1856, [BE|E] .08041
node N74: [E|B] 0.8565, [E|E] 0.07675, [BE|B] 0.03169
node N79: [A|E] 0.7513, [E|E] 0.1803, [AE|E] 0.03941
node N78: [E|E] 0.9908
node N106: [H|H] 0.4545, [H|A] .2035, [H|AH] 0.168, [EH|H] 0.0851, [AH|A] 0.03987
node N86: [H|H] 0.5859, [H|E] 0.2468, [H|EH] 0.04092, [EH|E] 0.04092, [H|A] 0.02872
node N105: [AH|A] 0.6071, [A|A] 0.2908, [A|AH] 0.07785
node N91: [A|AH] 0.5999, [A|A] 0.357
node N90: [H|A] 0.9171, [H|AH] 0.033
node N104: [A|A] 0.9063, [AH|A] 0.08951
node N96: [A|A] 0.8768, [A|AH] 0.1106
node N95: [A|A] 0.5464, [AH|A] 0.1097, [A|AH] 0.1097, [AH|H] 0.1097, [H|AH] 0.1097
node N103: [A|A] 0.9967
node N102: [A|A] 0.998
node N101: [A|A] 0.9974

* Split format: [left|right], where 'left' and 'right' are the ranges inherited by each descendant branch (on the printed tree, 'left' is the upper branch, and 'right' the lower branch).
