# Supplementary material for: In or Out-of-Madagascar?—Colonization Patterns for Large-Bodied Diving Beetles (Coleoptera: Dytiscidae)
Source: PLoS One. 2015 Mar 20;10(3):e0120777. doi: 10.1371/journal.pone.0120777 (PMC4368551; doi:10.1371/journal.pone.0120777)
Supplement: S9 Fig — (PDF) [file pone.0120777.s009.pdf]

Fg S9.

DEC model (Ree et al., 2005 and 2008)

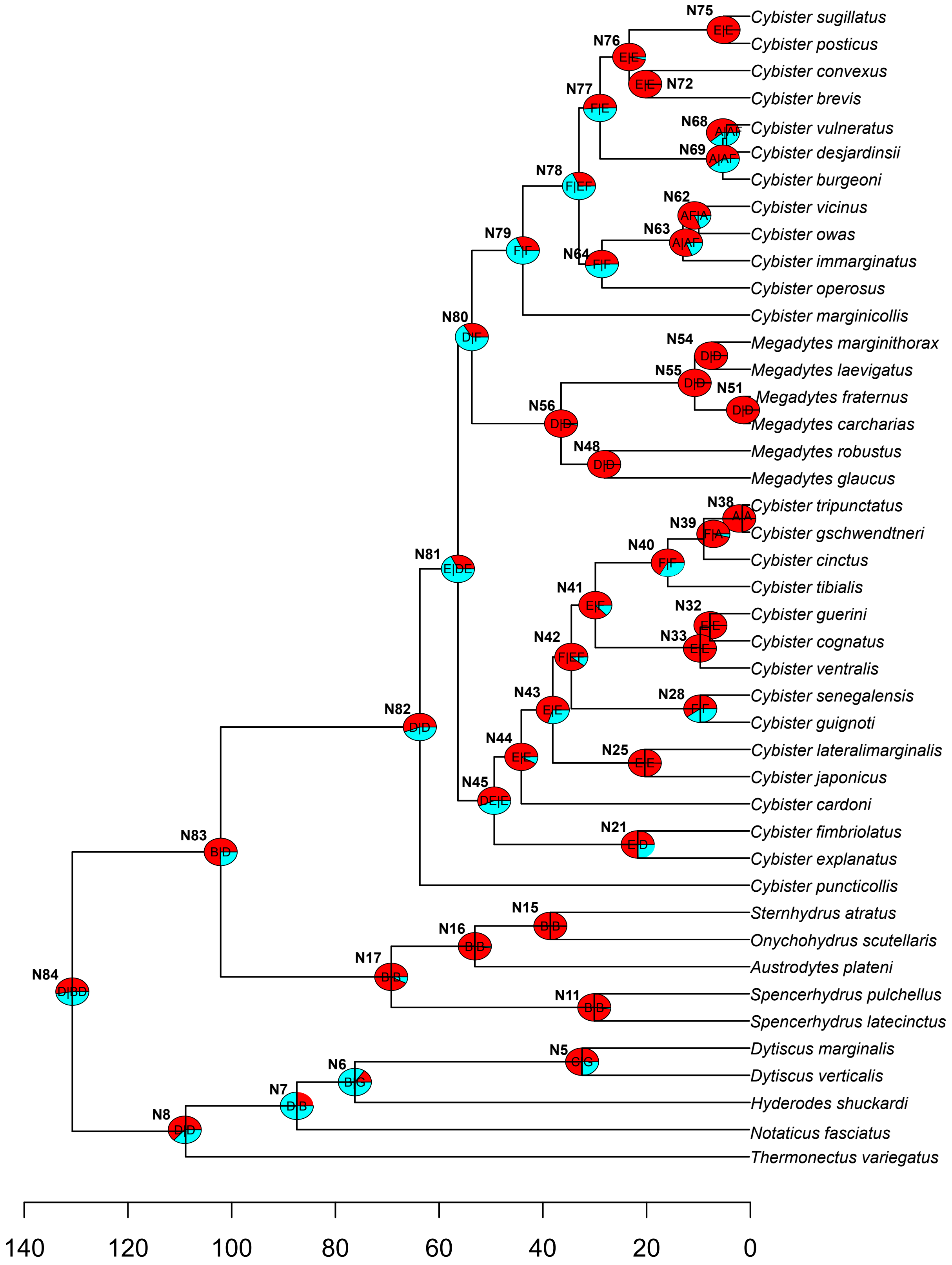

Legend:
A - Afrotropical
B - Australian
C - Nearctic
D - Neotropical
E - Oriental
F - Madagascar
G - Palearctic

Results:

node N84: [D|BD] 0.5257, [D|D] 0.1507, [BD|B] 0.05176, [CD|D] 0.04335, [DG|D] 0.04335
node N8: [D|D] 0.6365, [D|BD] 0.1516, [D|CD] 0.07484, [D|DG] 0.07478, [D|B] 0.01223
node N7: [D|B] 0.2556, [D|C] 0.1894, [D|G] 0.1893, [D|D] 0.1623, [BD|B] 0.03773
node N6: [B|C] 0.1546, [B|G] 0.1546, [C|C] 0.1348, [G|G] 0.1348, [B|B] 0.09812
node N5: [C|G] 0.7396, [C|C] 0.03214, [G|G] 0.03208, [C|G] 0.03179, [CG|G] 0.03176
node N83: [B|D] 0.7508, [D|D] 0.05452, [BD|D] 0.05264, [B|BD] 0.04282, [B|E] 0.02751
node N17: [B|B] 0.9235, [BD|B] 0.03443
node N11: [B|B] 0.9808
node N16: [B|B] 0.9812
node N15: [B|B] 0.9938
node N82: [D|D] 0.5587, [D|DE] 0.2616, [D|DF] 0.08896, [D|AD] 0.04887
node N81: [E|DE] 0.3195, [D|D] 0.2514, [D|DF] 0.1239, [DE|D] 0.1045, [D|AD] 0.08637
node N45: [DE|E] 0.5642, [E|E] 0.3542, [E|AF] 0.04147
node N21: [E|D] 0.7534, [E|C] 0.1995
node N44: [E|E] 0.918, [E|EF] 0.07265
node N43: [E|E] 0.7013, [E|EF] 0.2577
node N25: [E|E] 0.9979
node N42: [F|EF] 0.8879, [A|AE] 0.07907
node N28: [F|F] 0.6029, [F|AF] 0.3905
node N41: [E|F] 0.8688, [E|A] 0.08007, [EF|F] 0.02503
node N33: [E|E] 0.9952
node N32: [E|E] 0.9991
node N40: [F|F] 0.6707, [F|AF] 0.321
node N39: [F|A] 0.9599
node N38: [A|A] 0.997
node N80: [D|F] 0.3333, [D|E] 0.3328, [D|A] 0.2091, [D|D] 0.07404, [DF|F] 0.0111
node N56: [D|D] 0.9828
node N48: [D|D] 0.9951
node N55: [D|D] 0.9979
node N54: [D|D] 0.9996
node N79: [F|F] 0.3153, [F|EF] 0.2283, [A|A] 0.1887, [A|AE] 0.131, [AF|F] 0.02719
node N78: [F|EF] 0.314, [F|F] 0.2122, [A|AE] 0.1864, [A|A] 0.1184, [AF|A] 0.1124
node N64: [F|F] 0.5215, [F|AF] 0.2866, [F|A] 0.1305, [A|A] 0.05299
node N63: [A|AF] 0.8089, [A|A] 0.1864
node N62: [AF|A] 0.8108, [A|A] 0.1776
node N77: [F|E] 0.5137, [A|E] 0.4091, [A|A] 0.01718, [F|F] 0.01638
node N69: [A|AF] 0.6082, [A|A] 0.3896
node N68: [A|AF] 0.6154, [A|A] 0.3803
node N76: [E|E] 0.9682
node N72: [E|E] 0.9923
node N75: [E|E] 0.9976

* Split format: [left|right], where 'left' and 'right' are the ranges inherited by each descendant branch (on the printed tree, 'left' is the upper branch, and 'right' the lower branch).
