# Supplementary material for: In or Out-of-Madagascar?—Colonization Patterns for Large-Bodied Diving Beetles (Coleoptera: Dytiscidae)
Source: PLoS One. 2015 Mar 20;10(3):e0120777. doi: 10.1371/journal.pone.0120777 (PMC4368551; doi:10.1371/journal.pone.0120777)
Supplement: S1 Table — (PDF) [file pone.0120777.s010.pdf]

Genus	Species	Locality	COII	COI	H3	WNT
Cybister	brevis	Japan	DQ813773	DQ813669	DQ813735	DQ813708
Cybister	burgeoni	Namibia	DQ813670	DQ813670	DQ813736	DQ813709
Cybister	cardoni	India	DQ813774	DQ813671	DQ813737	DQ813710
Cybister	cinctus	Madagascar	DQ813777	DQ813674	DQ813740	DQ813713
Cybister	cognatus	India	DQ813775	DQ813672	DQ813738	DQ813711
Cybister	convexus	China	DQ813776	DQ813673	DQ813739	DQ813712
Cybister	desjardinsii	Mauritius	KP280096	KP280087	-	-
Cybister	explanatus	USA	DQ813778	DQ813675	DQ813741	DQ813714
Cybister	fimbriolatus	USA	DQ813779	DQ813676	DQ813742	AF392008
Cybister	gschwendtneri	Tanzania	DQ813780	DQ813677	DQ813743	DQ813715
Cybister	guerini	India	DQ813781	DQ813678	DQ813744	DQ813716
Cybister	guignoti	Madagascar	KP280097	KP280088	KP280105	-
Cybister	owas	Madagascar	KP280098	KP280089	KP280106	KP280112
Cybister	immarginatus	Ghana	DQ813782	DQ813679	DQ813745	DQ813717
Cybister	japonicus	Japan	DQ813783	DQ813680	DQ813746	DQ813718
Cybister	lateralimarginalis	Russia	DQ813784	DQ813681	DQ813747	-
Cybister	marginicollis	Madagascar	KP280094	KP280094	KP280110	KP280113
Cybister	operosus	Madagascar	KP280099	KP280090	KP280107	-
Cybister	posticus	India	DQ813786	DQ813683	DQ813749	DQ813720
Cybister	puncticollis	Bolivia	-	DQ813684	DQ813750	AF392009
Cybister	senegalensis	Madagascar	KP280100	KP280091	KP280108	-
Cybister	sugillatus	Hong Kong	DQ813788	DQ813686	DQ813752	DQ813722
Cybister	tibialis	Madagascar	KP280101	KP280092	-	-
Cybister	tripunctatus	Africa	KP280102	KP280093	KP280109	-
Cybister	ventralis	India	DQ813790	DQ813688	DQ813754	-
Cybister	vicinus	S.Africa	DQ813791	DQ813689	DQ813755	DQ813723
Cybister	vulneratus	Madagascar	KP280104	KP280095	KP280111	KP280114
Megadytes	carcharias	Peru	DQ813799	DQ813697	DQ813763	DQ813727
Megadytes	fraternus	Paraguay	DQ813800	DQ813698	DQ813764	DQ813728
Megadytes	glaucus	Argentina	DQ813801	DQ813699	DQ813765	DQ813729
Megadytes	laevigatus	Bolivia	DQ813802	DQ813700	DQ813766	-
Megadytes	marginithorax	Peru	-	DQ813701	DQ813767	DQ813730
Megadytes	robustus	Peru	DQ813803	DQ813702	DQ813768	DQ813731
Onychohydrus	scutellaris	Australia	DQ813805	DQ813704	DQ813770	DQ813733
Spencerhydrus	latecinctus	Australia	DQ813806	DQ813705	DQ813771	AF392043
Spencerhydrus	pulchellus	Australia	DQ813807	DQ813706	DQ813772	DQ813734
Sternhydrus	atratus	Australia	DQ813804	DQ813703	DQ813769	DQ813732
Hydaticus	arcuatus	Zambia	KP280130	KP280115	KP280145	-
Hydaticus	aruspex	New York	FJ796627	FJ796580	FJ796507	AF392019
Hydaticus	bihamatus	Australia	FJ796628	FJ796581	FJ796508	FJ796548
Hydaticus	bimarginatus	USA	FJ796629	FJ796582	-	FJ796549
Hydaticus	bivittatus	Madagascar	KP280140	KP280125	KP280154	KP280168
Hydaticus	bowringii	Japan	FJ796630	FJ796583	FJ796509	AF392020
Hydaticus	caffer	S.Africa	FJ796632	FJ796585	FJ796511	FJ796550
Hydaticus	capicola	S.Africa	FJ796636	FJ796589	FJ796514	FJ796552
Hydaticus	cinctipennis	USA	FJ796633	FJ796586	FJ796512	AF392021
Hydaticus	consanguineus	N. Caledonia	FJ796634	FJ796587	-	FJ796551
Hydaticus	continentalis	Russia	FJ796635	FJ796588	FJ796513	-
Hydaticus	dineutoides	Indonesia	FJ796637	FJ796590	FJ796515	-
Hydaticus	dorsiger	Madagascar	KP280141	KP280126	KP280155	KP280169
Hydaticus	dregei	Zimbabve	KP280142	KP280127	KP280156	-
Hydaticus	exclamationis	Madagascar	KP280146	KP280131	KP280146	KP280159
Hydaticus	fabricii	Philippines	FJ796640	FJ796593	FJ796518	AF392022
Hydaticus	flavolineatus	S.Africa	FJ796641	FJ796594	FJ796519	FJ796555
Hydaticus	galla	S.Africa	FJ796642	FJ796595	FJ796520	FJ796556
Hydaticus	grammicus	China	FJ796643	FJ796596	FJ796521	AF392023
Hydaticus	humeralis	Ghana	FJ796644	FJ796597	FJ796522	FJ796557
Hydaticus	kolbei	Madagascar	KP280143	KP280128	KP280157	KP280170
Hydaticus	intermedius	Madagascar	KP280132	KP280117	KP280147	KP280160
Hydaticus	lativittis	Ghana	FJ796648	FJ796601	FJ796526	FJ796561
Hydaticus	limnetes	Madagascar	KP280133	KP280118	KP280148	KP280161
Hydaticus	litiosus	India	FJ796646	FJ796599	FJ796524	FJ796559
Hydaticus	luczonicus	Vietnam	FJ796647	FJ796600	FJ796525	FJ796560
Hydaticus	madagascariensis	Madagascar	KP280134	KP280119	KP280149	KP280162
Hydaticus	major	Vietnam	FJ796649	FJ796602	FJ796527	AF392024
Hydaticus	matruelis	S.Africa	FJ796650	FJ796603	FJ796528	FJ796562
Hydaticus	nigrotaeniatus	Madagascar	FJ796651	FJ796604	-	KP280166
Hydaticus	orissaensis	India	FJ796652	FJ796605	FJ796529	FJ796563
Hydaticus	ornatus	Madagascar	KP280135	KP280120	KP280150	KP280163

Hydaticus	parallelus	Australia	FJ796654	FJ796607	FJ796530	AF392025
Hydaticus	petitii	Madagascar	KP280136	KP280121	KP280151	KP280164
Hydaticus	philippensis	Philippines	FJ796653	FJ796606	FJ796564	FJ796564
Hydaticus	pictus	United Arab Emirates	FJ796669	FJ796626	FJ796546	-
Hydaticus	quadrivittatus	Australia	-	FJ796609	-	FJ796566
Hydaticus	rhantoides	China	FJ796656	FJ796610	FJ796531	FJ796567
Hydaticus	ricinus	Vietnam	FJ796657	FJ796611	FJ796532	FJ796568
Hydaticus	rimosus	Costa Rica	FJ796658	FJ796612	FJ796533	FJ796569
Hydaticus	rivanolis	China	-	FJ796613	FJ796534	FJ796570
Hydaticus	saecularis	Madagascar	KP280144	KP280129	KP280158	KP280171
Hydaticus	satoi	China	FJ796659	FJ796614	FJ796535	FJ796571
Hydaticus	seminiger	Sweden	-	FJ796615	FJ796536	AF392026
Hydaticus	servillianus	Madagascar	KP280139	KP280124	KP280153	KP280167
Hydaticus	sobrinus	Madagascar	KP280137	KP280122	KP280152	KP280165
Hydaticus	speciosus	Ghana	FJ796660	FJ796616	FJ796537	FJ796572
Hydaticus	subfasciatus	Bolvia	FJ796662	FJ796618	FJ796539	FJ796574
Hydaticus	transversalis	Volgograd Obl.	FJ796663	FJ796619	FJ796540	FJ796575
Hydaticus	ugandaensis	Ghana	FJ796664	FJ796620	-	-
Hydaticus	ussheri	Ghana	FJ796665	FJ796621	FJ796541	FJ796576
Hydaticus	wattsi	Australia	FJ796667	FJ796623	FJ796543	FJ796578
Hydaticus	vittatus	India	FJ796666	FJ796622	FJ796542	FJ796577
Hydaticus	vitticollis	Zambia	KP280138	KP280123	-	-
Hydaticus	xanthomelas	Bolvia	FJ796668	FJ796624	FJ796544	AF392028
Dytiscus	marginalis	Sweden	DQ813793	DQ813691	DQ813757	DQ813725
Dytiscus	verticalis	New York	DQ813794	DQ813692	DQ813758	AF392012
Hyderodes	shuckardi	Australia	DQ813796	DQ813694	DQ813760	AF392018
Notaticus	fasciatus	Bolivia	-	FJ796625	FJ796545	-
Thermonectus	variegatus	Peru	KF978966	DQ431231	KF978998	KF979023
