# Supplementary material for: In or Out-of-Madagascar?—Colonization Patterns for Large-Bodied Diving Beetles (Coleoptera: Dytiscidae)
Source: PLoS One. 2015 Mar 20;10(3):e0120777. doi: 10.1371/journal.pone.0120777 (PMC4368551; doi:10.1371/journal.pone.0120777)
Supplement: S8 Fig — (PDF) [file pone.0120777.s008.pdf]

Fig S8.

Bayes-DIVA by Harris and Xiang (2009)

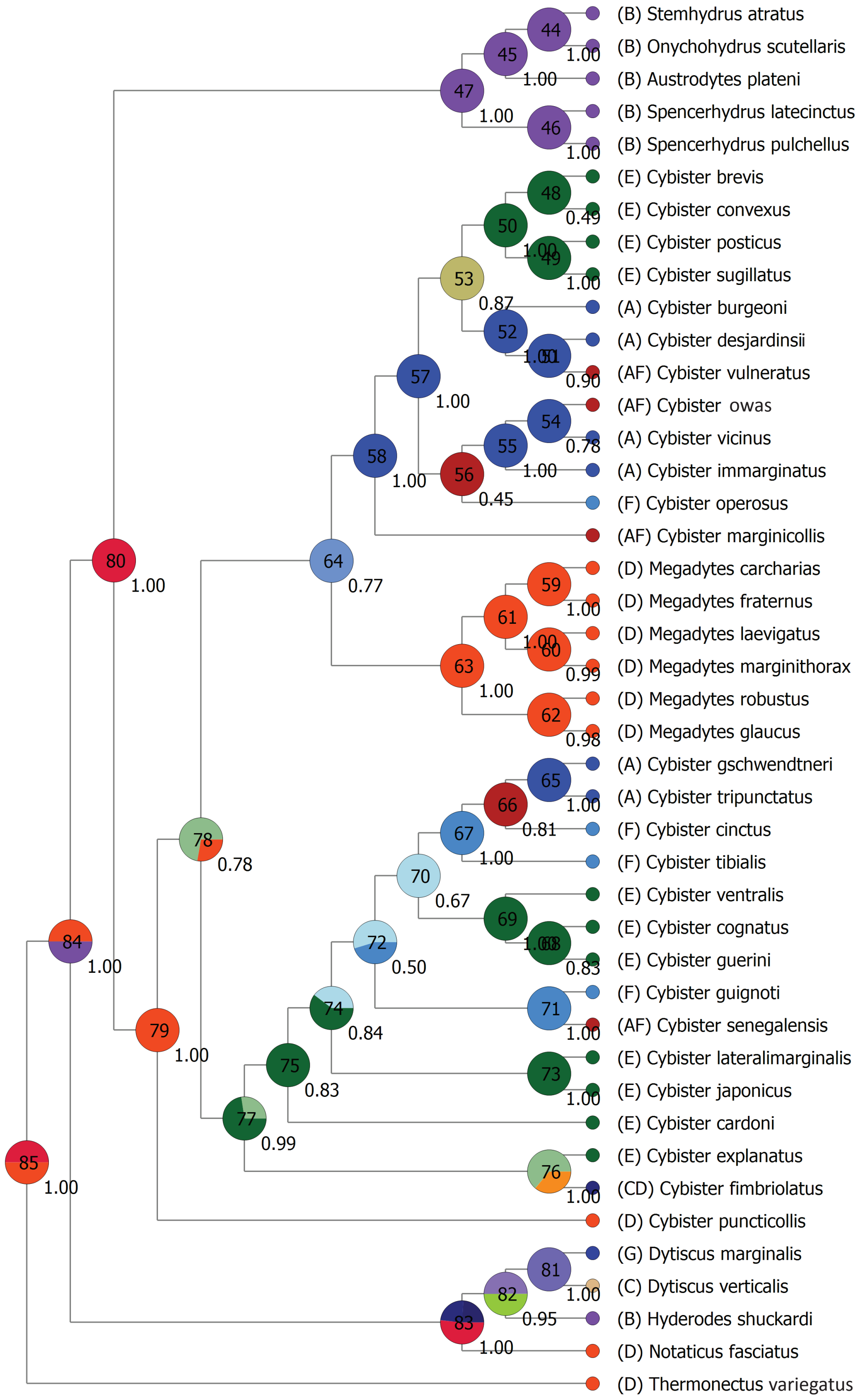

Legend:
A - Afrotropical
B - Australian
C - Nearctic
D - Neotropical
E - Oriental
F - Madagascar
G - Palearctic

Results:
optimal distributions at each node:
node 44 (anc. of terminals 42-39) (P=1.00): B 100.00
node 45 (anc. of terminals 42-1) (P=0.00): B 100.00
node 46 (anc. of terminals 40-41) (P=1.00): B 100.00
node 47 (anc. of terminals 42-41) (P=0.00): B 100.00
node 48 (anc. of terminals 2-7) (P=0.49): E 100.00
node 49 (anc. of terminals 19-22) (P=1.00): E 100.00
node 50 (anc. of terminals 2-22) (P=1.00): E 100.00
node 51 (anc. of terminals 8-37) (P=0.90): A 100.00
node 52 (anc. of terminals 3-37) (P=1.00): A 100.00
node 53 (anc. of terminals 2-37) (P=0.87): AE 100.00
node 54 (anc. of terminals 14-26) (P=0.78): A 100.00
node 55 (anc. of terminals 14-15) (P=1.00): A 100.00
node 56 (anc. of terminals 14-18) (P=0.45): AF 100.00
node 57 (anc. of terminals 2-18) (P=1.00): A 100.00
node 58 (anc. of terminals 2-36) (P=1.00): A 100.00
node 59 (anc. of terminals 30-31) (P=1.00): D 100.00
node 60 (anc. of terminals 33-34) (P=0.99): D 100.00
node 61 (anc. of terminals 30-34) (P=1.00): D 100.00
node 62 (anc. of terminals 35-32) (P=0.98): D 100.00
node 63 (anc. of terminals 30-32) (P=1.00): D 100.00
node 64 (anc. of terminals 2-32) (P=0.77): AD 100.00
node 65 (anc. of terminals 11-24) (P=1.00): A 100.00
node 66 (anc. of terminals 11-5) (P=0.81): AF 100.00
node 67 (anc. of terminals 11-23) (P=1.00): F 100.00
node 68 (anc. of terminals 6-12) (P=0.83): E 100.00
node 69 (anc. of terminals 25-12) (P=1.00): E 100.00
node 70 (anc. of terminals 11-12) (P=0.67): EF 100.00
node 71 (anc. of terminals 13-21) (P=1.00): F 100.00
node 72 (anc. of terminals 11-21) (P=0.50): F 45.3371
EF 54.6629
node 73 (anc. of terminals 17-16) (P=1.00): E 100.00
node 74 (anc. of terminals 11-16) (P=0.84): E 60.3181
EF 39.6819
node 75 (anc. of terminals 11-4) (P=0.83): E 100.00
node 76 (anc. of terminals 9-10) (P=1.00): CE 36.0389
DE 63.9611
node 77 (anc. of terminals 11-10) (P=0.99): E 72.6170
DE 27.3830
node 78 (anc. of terminals 2-10) (P=0.78): D 27.8093
DE 72.1907
node 79 (anc. of terminals 2-20) (P=1.00): D 100.00
node 80 (anc. of terminals 42-20) (P=0.00): BD 100.00
node 81 (anc. of terminals 28-27) (P=1.00): CG 100.00
node 82 (anc. of terminals 28-29) (P=0.95): BC 50.00
BG 50.00
node 83 (anc. of terminals 28-38) (P=1.00): BD 51.5771
CD 24.2114 DG 24.2114
node 84 (anc. of terminals 42-38) (P=0.00): B 50.00
D 50.00
node 85 (anc. of terminals 42-43) (P=1.00): D 50.00
BD 50.00
